# Crosstalk Between Metabolic Reprogramming and Epigenetic Modifications in Colorectal Cancer: Mechanisms and Clinical Applications

**DOI:** 10.3390/cimb47090751

**Published:** 2025-09-12

**Authors:** Yu-Hui Sun, Jing-Xian Zhang, Han-Shu Jin, Jin Huang

**Affiliations:** 1 Department of Gastroenterology, Graduate School of Nanjing Medical University, Nanjing 210000, China; 2023122414@stu.njmu.edu.cn (Y.-H.S.); 2024122072@stu.njmu.edu.cn (J.-X.Z.); jhs0729@stu.njmu.edu.cn (H.-S.J.); 2 Affiliated Changzhou No. 2 People’s Hospital of Nanjing Medical University, Changzhou 213161, China

**Keywords:** CRC, metabolic reprogramming, epigenetic modifications, tumor microenvironment, diagnosis, targeted therapy

## Abstract

Colorectal cancer (CRC) is one of the most common malignant tumors of the digestive tract in developing countries. It exhibits significant metabolic reprogramming and epigenetic abnormalities during its development. These two changes interact at the molecular level and jointly promote the progression of tumor cells. Cancer cells reprogram metabolites such as glucose, glutamine, and lipids to meet their energy and biological substrate requirements for survival. Concurrently, abnormalities in epigenetic modifications drive imbalances in gene expression and sustain the malignant phenotype. More importantly, metabolites can serve as substrates or cofactors for epigenetic enzymes, and changes in metabolic status can induce epigenetic remodeling. Correspondingly, epigenetic mechanisms regulate the transcription and function of metabolism-related genes, leading to adaptive alterations in tumor metabolic pathways. This review systematically summarizes the characteristics of major metabolic pathway reprogramming and the mechanisms underlying key epigenetic abnormalities in CRC. Furthermore, it elaborates on the mechanisms of their mutual influence in signaling pathways, key factors, immunometabolism, and the tumor microenvironment. It also discusses recent advances in novel diagnostic technologies (such as multi-omics integrated diagnostics) and therapeutic strategies (including targeting metabolism, epigenetic therapy, and combination therapies). In the future, research focusing on the interaction between metabolic reprogramming and epigenetics will provide new insights and targets for the early diagnosis and precision treatment of CRC.

## 1. Introduction

CRC ranks as the third most common and second deadliest malignancy globally, posing a severe threat to public health worldwide [1]. Its development is influenced by multiple factors, including genetic mutations, lifestyle, and environment [2,3]. Among these, metabolic reprogramming and aberrant epigenetic modifications in tumor cells have been recognized as two critical hallmarks of cancer initiation and progression [4]. Metabolic reprogramming denotes the rewiring of nutrient utilization to sustain malignant proliferation. In CRC, aerobic glycolysis generates lactate while diverting glycolytic intermediates into nucleotide, amino-acid, and lipid biosynthesis to supply ATP, reducing power, and biomass [5,6]. Epigenetic modifications are heritable changes in gene regulation without alteration of the DNA sequence, encompassing DNA methylation, histone post-translational modifications, and noncoding-RNA–mediated control [7,8]. In CRC, epigenetic remodeling reshapes the expression of metabolic and immune-evasion genes, thereby modulating the tumor microenvironment; these changes establish stable aberrant transcriptional states that can influence neighboring cells [9].

Notably, metabolic status and epigenetic regulation do not exist in isolation. Metabolites (e.g., acetyl-CoA, αketoglutarate) can serve as substrates for epigenetic enzymes (e.g., histone acetyltransferases, deacetylases, DNA methyltransferases, demethylases) [10]. Alterations in cellular metabolic pathways influence epigenetic modification levels through these metabolic mediators. For instance, metabolic reprogramming leading to the accumulation or deficiency of specific metabolic intermediates can directly regulate modifications such as DNA/histone methylation and acetylation [11]. This indicates that cancer cells achieve adaptive remodeling of gene expression through metabolic reprogramming and epigenetic modification axis. In addition, epigenetic mechanisms also participate in regulating the expression of enzymes and metabolic pathway genes related to metabolism, thereby determining the metabolic program of tumor cells [12,13]. Thus, metabolic reprogramming and epigenetic dysregulation form a complex bidirectional regulatory network in CRC, profoundly impacting tumor initiation, progression, and therapeutic response [14].

This review aims to summarize the key features of metabolic reprogramming and epigenetic modifications in CRC, as well as their cross-regulatory mechanisms. In the section on metabolic reprogramming, we will discuss in detail the alterations and regulatory mechanisms of glycolysis, glutamine metabolism, lipid metabolism, and other pathways in CRC. In the epigenetic modifications section, we will introduce abnormalities in DNA methylation, histone modifications, noncoding RNA, and other aspects in CRC. Subsequently, in the interaction mechanisms section, we will elaborate at the molecular level on how metabolites influence epigenetic enzyme activity and gene expression, how epigenetic regulation reshapes cellular metabolism, and the role of the metabolism–epigenetics axis in signaling pathways, immunometabolism, and the tumor microenvironment. Next, we will also discuss recent advances in novel diagnostic approaches for CRC based on cutting-edge omics technologies and emerging therapeutic strategies. Ultimately, research focused on metabolism and epigenetic modifications will drive progress in the diagnosis and precision treatment of CRC.

## 2. Method

We used ClinicalTrials.gov as the sole data source. The search covered from database inception to 31 August 2025, with the last search performed on 31 August 2025. Terms related to colorectal cancer and metabolic pathways were combined in the Condition/Disease and Other terms fields, including but not limited to: colorectal cancer, metabolism, metabolic, glycolysis, oxidative phosphorylation, fatty acid, lipid metabolism, one-carbon, serine glycine, folate, purine, pyrimidine, glutaminase, lactate dehydrogenase, as well as representative drug or intervention names. The platform’s automatic MeSH mapping was enabled by default, and all returned records were manually verified and cleaned to reduce noise and remove duplicates.

Prespecified inclusion criteria were interventional clinical trials in adults with colorectal cancer in which the intervention directly targeted a metabolic pathway or clearly modulated a metabolic phenotype, either as monotherapy or in combination with standard treatments, and that reported at least one clinical efficacy endpoint or a prespecified biomarker or pharmacodynamic endpoint together with safety. Exclusion criteria were observational or purely translational or mechanistic studies, mixed-tumor trials without extractable colorectal cancer data, interventions not pertaining to metabolic pathways, duplicate entries, or nonprimary records of the same trial. Two reviewers independently screened titles and records, conducted record-level assessment, and extracted the NCT identifier, study title, patient population, intervention and comparator, trial phase, primary endpoint, key outcome notes, and trial status. Discrepancies were resolved by consensus with a third reviewer. When multiple entries existed for the same trial, the most recent and most complete record was retained.

## 3. Metabolic Reprogramming in CRC

### 3.1. Glycolytic Reprogramming

Even under aerobic conditions, tumor cells preferentially generate energy through glycolysis. This unique metabolic pattern is termed metabolic reprogramming in cancer cells, also known as the Warburg effect. In CRC, substantial amounts of glucose are taken up by tumor cells despite intact mitochondrial oxidative phosphorylation, leading to massive lactate production. The Warburg effect not only fulfills the energy and metabolic intermediate demands for rapid tumor growth but also promotes tumor cell invasion and metastasis [15]. Compared to normal intestinal mucosal tissue, critical alterations occur in multiple metabolic pathways in CRC, resulting in proto-oncogene activation and tumor suppressor gene inactivation. Studies reveal that approximately 40% of CRC cases exhibit significant K-RAS gene mutations [16]. K-RAS mutations markedly increase the expression of glucose transporter GLUT1, enhancing glucose uptake and glycolytic flux. Mutations in the tumor suppressor gene p53 occur in over 40% of CRC cases. Loss of wild-type p53 function abolishes its inhibitory effect on glycolysis, promoting greater dependence on glycolysis for energy supply. In p53 wild-type CRC cells, glycolysis contributes approximately 40% to ATP production, whereas this proportion rises to about 66% in p53 mutant cells [17]. Research by Jing et al. found that NCAPD3 is overexpressed in CRC and significantly correlates with poor prognosis. It drives glycolytic reprogramming through dual synergistic mechanisms [18]. Constitutive oncogenic signaling couples glycolytic flux to growth demands in CRC. KRAS activation, frequently cooperating with near-normoxic HIF-1α stabilization, increases glucose uptake and aerobic glycolysis by elevating SLC2A1/GLUT1, HK2, PFKFB3, PKM2 and LDHA, with consequent lactate export that supports redox balance and microenvironmental adaptation [16,19]. These transcriptional and post-transcriptional programs correlate with aggressive phenotypes and therapy resistance in CRC models and patient samples. In parallel, Wnt/β-catenin activity sustains MYC-dependent expression of glycolytic and lactate-transport modules and dynamically tunes the balance between oxidative phosphorylation and glycolysis in response to nutrient and oxygen availability [20].

**Figure 1:**
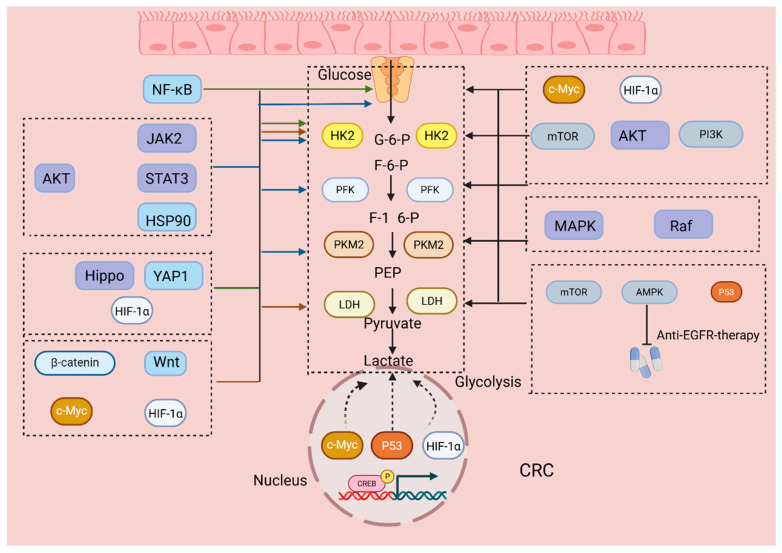
Overview of Glycolysis and Key Signaling Inputs in Colorectal Cancer. Glucose enters via GLUT and flows through the glycolytic core (HK2→PFK→PKM2→LDH) to pyruvate and lactate. Representative pathways and factors modulating glycolysis are shown: PI3K–AKT–mTOR, MAPK–Raf, the NF-κB axis, Hippo–YAP1, Wnt/β-catenin, and HIF-1α, c-Myc, P53–AMPK.

On one hand, NCAPD3 binds to c-Myc and recruits it to the promoters of glycolytic regulators such as GLUT1, and LDHA, enhancing glycolysis in CRC cells. On the other hand, it promotes E2F1 expression, enabling E2F1 to occupy the promoters of PDK1, thereby inhibiting PDH activity and impairing the TCA cycle. This metabolic shift reinforces the Warburg effect, supporting CRC cell proliferation and growth. Additionally, research by Padder et al. demonstrated that RAFV600E mutation causes CRC cells to upregulate PDK1 and activate DRP1, inducing excessive mitochondrial fission and forming highly fragmented mitochondrial morphology [21]. This drives glycolytic metabolism, increases ATP and NADPH supply, and enhances clonogenicity and metastatic capacity. Similarly, Guan et al. found that under chronic stress conditions, sustained adrenaline release activates β2-adrenergic receptors, triggering PKA-mediated CREB1 phosphorylation. Phosphorylated CREB1 translocates to the nucleus and upregulates transcription of key glycolytic enzymes such as HK2, and PFKP. This remodels tumor energy metabolism, enhances glycolysis in CRC cells, and provides additional ATP and metabolic intermediates, thereby accelerating proliferation and promoting tumor growth in mice [22]. Studies indicate that highly expressed miR-27a in CRC suppresses AMPK and activates mTOR signaling, reducing oxidative phosphorylation and overall mitochondrial function. This forces tumor cells to favor aerobic glycolysis [23]. These pathways supply substrates and energy for CRC cells to synthesize macromolecules, sustaining proliferation and survival while diminishing chemotherapy sensitivity. These alterations establish glycolytic reprogramming as the critical metabolic foundation enabling CRC cells to adapt to the tumor environment, sustain proliferation, and acquire invasiveness (Figure 1).

Current evidence supports the shaping role of KRAS and p53 status on glycolytic flux and the fractional ATP contribution from glycolysis; however, most data derive from cell lines and small tissue series. Estimates of ATP partitioning are highly contingent on culture conditions, carbon sources, and oxygen tension, and cross-laboratory reproducibility remains to be established. The study showing that NCAPD3 drives glycolysis and pyruvate routing via dual c-Myc and E2F1 pathways is mechanistically clear yet largely limited to in vitro and murine systems, with no population-level external validation linking high NCAPD3 to metabolic phenotypes or drug response. The BRAF V600E–PDK1–DRP1 axis that induces mitochondrial fission and biases toward glycolysis highlights coupling between metabolism and mitochondrial dynamics, but it depends heavily on a specific mutation background and may not generalize to wild-type or other mutational contexts. The adrenaline–β2-adrenergic receptor–CREB1 pathway under chronic stress and the miR-27a–AMPK–mTOR pathway illustrate endocrine and noncoding-RNA inputs that reprogram metabolism upstream, yet the intensity and duration of stress in animal models are not fully comparable to clinical scenarios.

Next steps should include prospective cohorts that integrate isotope tracing with tissue and liquid-biopsy metabolic fingerprints, stratified by genotype and microenvironment, and pharmacodynamic validation of candidate nodes linked to clinical outcomes in human samples.

### 3.2. Amino Acid Metabolic Reprogramming

Besides glucose, tumor cells exhibit heavy reliance on amino acids as metabolites. Glutamine, the most abundant amino acid in the human body, is consumed at the fastest rate among amino acids in cancer cells and is hailed as the “second fuel” [24]. CRC cell growth is highly dependent on glutamine; its absence in the culture medium severely inhibits proliferation. After entering cells via glutamine transporter ASCT2, glutamine is hydrolyzed into glutamate and ammonia by glutaminase (GLS) in the cytoplasm [25]. Glutamate is subsequently converted to α-ketoglutarate to enter the tricarboxylic acid (TCA) cycle, providing cycle intermediates and energy. Research by Han et al. revealed that increased DNA copy number in CRC leads to overexpression of the m^6A “reader” YTHDF1 [26]. YTHDF1 enhances the translation efficiency of GID8 mRNA by recognizing its m^6A site, elevating GID8 levels. Highly expressed GID8 maintains the transcription and protein stability of SLC1A3 and GLS, increasing glutamine uptake and catabolism in tumor cells to meet the carbon and energy demands for rapid proliferation and metastasis. Inhibiting GID8 significantly impairs cell proliferation, invasion, and tumor growth in mouse models. Additionally, Hua and colleagues similarly discovered that CEMIP is markedly overexpressed in CRC. CEMIP acts as a novel adaptor protein for OGT, facilitating O-GlcNAcylation of β-catenin and promoting its nuclear translocation [27]. Intriguingly, nuclear β-catenin in turn enhances CEMIP transcription, forming a self-amplifying positive feedback loop. Concurrently, nuclear β-catenin upregulates GLS1 and glutamine transporters SLC38A2, remodeling glutamine metabolism to provide energy and biosynthetic substrates for cancer cell metastasis. Beyond glutamine, other amino acids undergo metabolic reprogramming. Lin et al. demonstrated significant cysteine enrichment in CRC tissues. Hypoxia-induced ROS and endoplasmic reticulum stress activate transcription factor ATF4, cooperatively upregulating cysteine transporters SLC1A4/SLC1A5 and cystine transporters SLC7A11/SLC3A2 [28]. This ensures simultaneous uptake of cysteine and cystine by tumor cells to maintain intracellular cysteine levels. Meanwhile, GSS overexpression enhances cysteine flux toward reduced glutathione, conferring antioxidant and growth advantages. Under serine-deprived stress conditions, CRC cells activate the ERK1/2-pELK1 signaling axis, upregulating transcription factor FOXC1. Overexpressed FOXC1 then activates key serine synthesis pathway enzymes (PHGDH, PSAT1, PSPH), restoring intracellular serine supply to sustain proliferation [29]. This process improves 5-FU tolerance by enhancing purine synthesis and DNA damage repair. In KRAS-mutant CRC, activated PI3K-AKT-mTOR signaling upregulates asparagine synthetase (ASNS), driving massive conversion of aspartate to asparagine. This pathway simultaneously reduces aspartate and elevates asparagine. KRAS- and Wnt/β-catenin–MYC–driven programs reconfigure nitrogen and carbon sourcing from amino acids to sustain biosynthesis and redox homeostasis. Glutamine addiction is reinforced by MYC-induced SLC1A5 and GLS1, feeding anaplerosis and NADPH production [30]. When glutamine becomes limiting, KRAS-mutant CRC cells adapt by upregulating asparagine synthetase (ASNS) to preserve protein synthesis and survival [31]. Converging signals also elevate the serine–glycine–one-carbon axis, with increased PHGDH, PSAT1 and SHMT2 expression that fuels nucleotide synthesis and redox buffering. Emerging CRC data further link SGOC enzymes to chemoresistance [32]. Essential amino-acid acquisition via LAT1 (SLC7A5) supports mTORC1 activation and growth and shows prognostic relevance in CRC, suggesting tractable nodes at transporters and transaminases alongside glutaminase and one-carbon enzymes.

Sufficient asparagine sustains cell growth and survival during glutamine scarcity, enabling tumors to evade nutrient limitation. Further experiments confirmed that inhibiting ASNS or combining L-asparaginase with rapamycin significantly blocks KRAS-mutant tumor proliferation, indicating ASNS-driven asparagine synthesis is key to metabolic adaptation and therapy resistance in KRAS-mutant CRC [33].

The YTHDF1–GID8 axis that sustains SLC1A3 and GLS expression and drives glutamine uptake and catabolism is well supported, but the evidence rests mainly on gain- and loss-of-function assays and spheroid models, with limited quantitative causality between intratumoral glutamine flux and proliferative demand. CEMIP acting as an OGT adaptor to promote β-catenin nuclear translocation and upregulate GLS1 and SLC38A2 suggests positive feedback between glycosaminoglycan biology and Wnt–glutamine circuitry, although epithelial–mesenchymal heterogeneity and metastatic-site differences have not been systematically assessed. Coordinated cysteine and cystine uptake together with GSS-driven glutathione synthesis explains anti-apoptotic phenotypes, but prospective evidence linking these features to chemotherapy response in patients is lacking. The ERK–pELK1–FOXC1 axis that induces serine biosynthesis and 5-fluorouracil tolerance is temporally coherent, yet requires confirmation of flux changes through isotope tracing and intratumoral biomarkers. In KRAS-mutant disease, ASNS upregulation underpins asparagine dependence; combinations of L-asparaginase with mTOR inhibition are rational but will need biomarker-guided early-phase trials to delineate toxicity and the eligible population. Overall, the causal chain for amino-acid reprogramming is largely in place, and the priority is to connect flux readouts, genotype stratification, and clinical endpoints within a single prospective framework.

### 3.3. Lipid Metabolic Reprogramming

In normal cells, fatty acid and cholesterol metabolism remain at relatively low levels, whereas tumor cells frequently exhibit aberrant lipid synthesis and catabolism to meet demands for membrane composition and signaling molecule generation. In CRC, the fatty acid synthesis pathway is often markedly enhanced [34]. Key enzymes such as ACLY, ACC, and FASN are highly expressed in CRC tissues. These enzymes channel carbon sources like glucose and glutamine via the TCA cycle and acetyl-CoA generation pathways into de novo fatty acid synthesis for building membrane phospholipids, cholesterol esters, and signaling molecules [35]. Research by Dong et al. showed that FUT2 is overexpressed in CRC and correlates with malignant phenotypes and enhanced fatty acid metabolism [36]. Through fucosylation, FUT2 facilitates nuclear translocation of YAP1 and stabilizes mSREBP-1, thereby amplifying SREBP-1’s transcriptional activation of fatty acid synthase genes. This enhances glucose uptake and de novo fatty acid synthesis, providing sustained energy and membrane lipid precursors to promote tumor proliferation and metastasis. Knocking down FUT2 disrupts this metabolic reprogramming and significantly suppresses tumor growth and invasion. Additionally, Zhao and colleagues found that HMGA1 substantially increases the activity of transcription factor SREBP1, elevating the transcription and protein levels of fatty acid synthase FASN. A high-fat diet further amplifies this lipid metabolic pathway’s pro-tumor effect [37]. Conversely, FASN inhibitors like orlistat markedly slow tumor growth in HMGA1-overexpressing mouse models. Similarly, FASN enhances the activity of transcription factor SP1, increasing expression of phospholipase PLA2G4B, which promotes phosphatidylcholine (PC) synthesis and accumulation [38]. PC enrichment not only supplies membrane lipid precursors and signaling molecules to CRC cells but also supports their proliferation, migration, and invasion. Silencing FASN reduces PC levels, blocks CRC cell proliferation and lung metastasis, and restores NK cell-mediated immune effects. Research by Yang et al. revealed that mitochondrial citrate transporter SLC25A1 is significantly upregulated in CRC tissues and closely associated with poor patient survival. SLC25A1 promotes tumor growth through a dual metabolic strategy: under nutrient-rich conditions, it exports mitochondrial citrate to the cytosol to boost acetyl-CoA production and enhance de novo synthesis of fatty acids and phospholipids, providing membrane lipids and energy for rapid proliferation; during energy stress or nutrient deprivation, it increases TCA cycle substrate supply and electron transport efficiency in mitochondria, strengthening oxidative phosphorylation to maintain ATP supply and resist stress-induced apoptosis. Knocking down SLC25A1 induces G1/S cell cycle arrest and apoptosis, significantly suppressing tumor growth in vitro and in vivo [39]. Lipid metabolic reprogramming also plays a key role in CRC metastasis. In CRC liver metastasis, the Lyn/RUVBL1 complex drives metastasis through two interactive pathways [40]. First, the complex remodels the 3D chromatin structure of the TRIB3 promoter and enhances Pol II occupancy, elevating TRIB3 expression to stabilize β-catenin. This subsequently upregulates MMP9 and VEGF to promote proliferation and migration. Second, the complex increases FOXA1 binding accessibility to the COX2 promoter and elevates H3K27ac levels, activating COX2-PGE2 synthesis and intensifying arachidonic acid metabolism. PGE2 further promotes β-catenin nuclear translocation and synergistically enhances MMP9 and VEGF expression. These dual pathways couple epigenetic remodeling with metabolic reprogramming, accelerating CRC metastasis to the liver. Furthermore, the PI3K/AKT/mTOR axis promotes de novo lipogenesis and membrane biogenesis through SREBP1 activation, which transcriptionally upregulates ACLY, ACACA and FASN, thereby diverting citrate toward fatty-acid synthesis while restraining fatty-acid oxidation and preserving acetyl-CoA for anabolism [41]. This lipogenic rewiring interfaces with redox control and cell-death susceptibility; hyperactive PI3K/AKT/mTOR signaling maintains SREBP1/SCD1-driven monounsaturated fatty acids that blunt lipid peroxidation and confer resistance to ferroptosis, highlighting therapeutic vulnerabilities to FASN, ACLY or SREBP1 pathway inhibition and to rational ferroptosis-inducing strategies [42].

Data implicating FUT2-mediated fucosylation in YAP1 nuclear translocation and mSREBP-1 stabilization identify a point of intersection between glycosylation and lipogenesis, yet the clinical prevalence and prognostic value of FUT2-high subsets require large external validation. The HMGA1–SREBP1–FASN axis is amplified by high-fat diet, indicating lifestyle–target interplay, although the translational fidelity of diet models and the selectivity of FASN inhibitors warrant careful appraisal. The FASN–SP1–PLA2G4B pathway linking phospholipid metabolism to NK-cell suppression is provocative, but causal directionality in the immune microenvironment needs spatial omics and functional blockade for confirmation. SLC25A1’s switching between cytosolic lipogenesis and mitochondrial oxidative support across nutrient states underscores metabolic plasticity as a driver of drug tolerance. The Lyn–RUVBL1 complex promoting liver metastasis through TRIB3 stabilization and COX2–PGE2 activation connects chromatin remodeling to lipid signaling, yet combination strategies with anti-inflammatory or lipid-metabolism inhibitors remain preclinical. Incorporating targeted lipidomics and spatial metabolic imaging into clinical specimens would help quantify pathway activity and metastatic risk, while pharmacodynamic and immunologic composite endpoints could clarify the net benefit and safety margins of lipid-pathway inhibition.

### 3.4. Reprogramming of Other Metabolic Pathways

Beyond the above pathways, one-carbon metabolism and nucleotide metabolism are also dysregulated in CRC. Nucleotide availability constitutes a metabolic bottleneck for proliferation, drug resistance, and evolutionary progression in colorectal cancer, and tumors sustain the nucleotide pool together with mitochondrial function through multiple coordinated axes. At the level of mitochondrial one-carbon metabolism, SHMT2 converts serine into one-carbon units to drive purine synthesis and stabilize the dNTP pool, thereby buffering 5-fluorouracil–induced replication and repair stress [43]. Genetic or pharmacologic inhibition of SHMT2 amplifies DNA breaks and cell-cycle arrest, whereas rescue by exogenous nucleosides indicates a causal linkage to nucleotide supply. In the dimension of iron homeostasis, tumor epithelium ectopically activates hepcidin to internalize the iron exporter ferroportin, producing intracellular iron accumulation that is preferentially funneled into nucleotide biosynthesis. Iron chelation concomitantly impairs mitochondrial function and nucleotide production, and mouse genetics together with upstream HIF-2α regulation connect hypoxia to iron–nucleotide coupling [44]. With respect to carbon-flux allocation, SIRT5 de-succinylates and activates transketolase (TKT), augmenting the non-oxidative pentose-phosphate pathway to generate ribose-5-phosphate and maintain purine/pyrimidine precursors and genomic integrity. SIRT5 silencing lowers nucleotide levels and increases DNA-damage signaling, a phenotype partially reversible by nucleoside supplementation [45]. Spatial and single-cell analyses further provide histologic context by identifying a nucleotide-metabolism–high epithelial subset that resides adjacent to fibroblasts and engages collagen–integrin adhesion (for example, COL1A1/COL1A2–ITGB1), nominating NME1 as a key gene, and yielding a prognostic score that stratifies survival and predicts immunotherapy response [46]. Collectively, the SHMT2–one-carbon supply, hepcidin-mediated iron redistribution, and SIRT5/TKT-driven non-oxidative PPP form three pillars of nucleotide maintenance which, together with matrix-anchored interactions in tissue space, underpin malignant behavior and suggest translational strategies centered on metabolic-node inhibition, exploitation of nucleotide-metabolism vulnerabilities, and microenvironmental intervention.

S-adenosylmethionine (SAM), generated by folate-mediated methyl cycles, acts as a methyl donor and bridges carbon metabolism with epigenetic regulation [47]. Studies show that cancer stem cells heavily depend on exogenous methionine due to high SAM consumption [48]. Inhibiting methionine metabolism enzymes (e.g., MAT2A) reduces histone methylation and suppresses tumor proliferation. Dietary folate levels also influence methyl metabolism in colorectal mucosa, folate deficiency lowers SAM and causes global DNA hypomethylation, increasing CRC risk [49]. However, findings on folate’s impact are conflicting some mouse models indicate folate deficiency inhibits CRC growth without significantly reducing DNA methylation, suggesting additional regulatory factors. Specifically, in premalignant or very early stages, restricting one-carbon flux transiently suppresses nucleic-acid synthesis, making it difficult for highly proliferative adenomatous cells to complete replication and thereby exerting an inhibitory effect. Once a tumor is established, chronic folate insufficiency lowers thymidylate-synthesis efficiency and induces uracil misincorporation, increasing DNA breaks and chromosomal instability that accelerate clonal selection and aggressive evolution. Folate supplementation can improve genome stability and mucosal repair in folate-deficient populations, but in the setting of existing lesions the added supply of nucleotides and methyl donors may confer a growth advantage to cancer cells, yielding a time-dependent biphasic effect. The coupling between metabolism and epigenetics further amplifies this context dependence. The folate cycle sets the intracellular balance of S-adenosylmethionine and S-adenosylhomocysteine, thereby governing the activity of DNA methyltransferases and histone methyltransferases. Modest elevations of S-adenosylmethionine help preserve the epigenetic integrity of tumor-suppressor promoters, whereas abundant methyl-donor availability within a pro-oncogenic signaling milieu may stabilize unfavorable chromatin states. Differential contributions of carbon sources to acetyl-CoA and one-carbon flux mean that identical folate levels can produce distinct epigenetic readouts across microenvironments. Tumor genotype further modulates directionality, as KRAS or p53 alterations shift dependency on thymidylate and purine synthesis, altering the metabolic benefits and risks of folate manipulation. Folate and short-chain fatty acids produced by the microbiota modify local substrate availability in the intestinal epithelium, decoupling dietary folate intake from tissue-level one-carbon flux.

**Figure 2:**
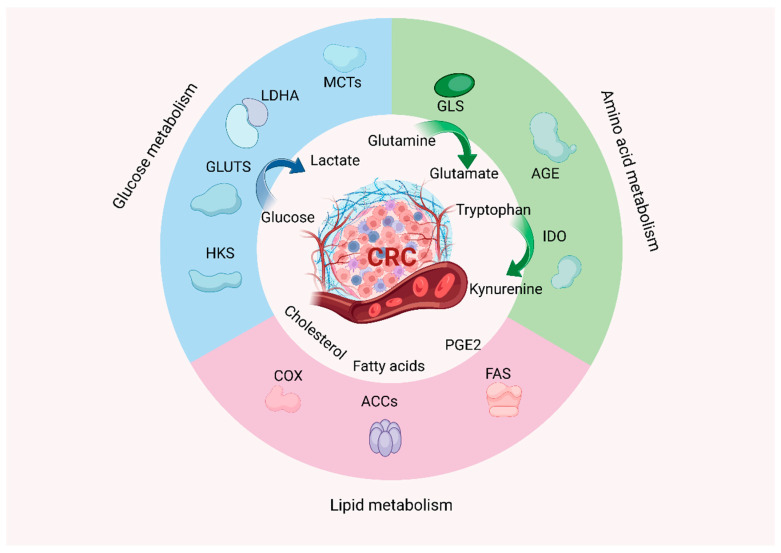
Metabolic reprogramming in CRC cells. CRC cells utilize glucose metabolism reprogramming to generate more lactic acid, thereby inducing an acidic microenvironment and generating substrates for epigenetic modifications. At the same time, they utilize fatty acid and amino acid metabolism reprogramming to promote tumor cell generation, invasion, and metastasis. The figure was created with BioRender.com.

Moreover, CRC cells prefer de novo nucleotide synthesis over the salvage pathway used by normal cells. Integrated analyses reveal significantly elevated activity of purine/pyrimidine de novo synthesis enzymes in CRC tissues [50]. Metabolic signaling and protein synthesis regulation are also hyperactive, for instance, PI3K/mTOR pathway overactivation in CRC not only promotes glycolysis but also upregulates key translation factors for protein synthesis. In summary, CRC cells achieve rebalanced energy metabolism and biosynthesis through comprehensive metabolic reprogramming, providing the material basis for tumor growth, invasion, and environmental adaptation (Figure 2).

## 4. Epigenetic Modifications

### 4.1. DNA Methylation

DNA methyltransferases add methyl groups to the 5′ carbon atom of cytosine in CpG dinucleotides via covalent bonds, which is the most typical DNA methylation process, forming 5-methylcytosine (5mC) [51]. In normal cells, high-density CpG islands in promoter regions typically remain unmethylated to permit gene transcription, while repetitive sequences scattered throughout the genome are highly methylated to maintain genomic stability. Generally, a hypermethylated state indicates gene suppression or silencing, whereas a hypomethylated state indicates gene activation [52]. Hypermethylation is a key mechanism in CRC formation, particularly within CpG islands of tumor suppressor genes. Studies reveal that genes such as ADHFE1, CNN1, NR3C1, SFRP, ITGA4, and ADAMTS14 undergo hypermethylation in CRC, leading to their inactivation [53,54]. These genes participate in critical processes like signal transduction, cell cycle regulation, and angiogenesis; their silencing promotes CRC development. Hypermethylation of the ITGA4 promoter region is significantly expressed in tumor samples, indicating its association with poor prognosis in CRC patients. Additionally, approximately 15% to 20% of CRC cases exhibit the CIMP, characterized by aberrant methylation of numerous gene promoters [55]. Hypermethylation of the DNA mismatch repair gene MLH1 promoter inactivates MLH1, leading to sporadic MSI-H CRC [56]. Furthermore, tumor suppressor genes such as APC, RASSF1A, and the SFRP family are often transcriptionally silenced in CRC due to promoter methylation. DNA methylation status is regulated by active demethylation mediated by TET family dioxygenases. TET enzymes oxidize 5mC to intermediates like 5-hydroxymethylcytosine (5hmC), ultimately achieving cytosine demethylation. In CRC, TET2/3 function is often impaired, causing a significant decline in 5hmC levels. Studies report that 5hmC content is markedly reduced in primary CRC tissues compared to normal mucosa and correlates with poor patient survival. Research by Kawaguchi et al. found that the high expression of FABP5 in metastatic CRC cells stems from promoter demethylation, and this hypomethylated state is closely linked to the expression pattern of DNMT3B splice variants [57]. The demethylated promoter can be bound by NF-κB, driving elevated FABP5 transcription. Subsequently, FABP5 further enhances NF-κB activity by promoting IL-8 production, forming a DNA methylation-dependent NF-κB/FABP5 positive feedback loop that persistently activates NF-κB signaling and accelerates CRC progression. Low methylation, including chromosomal instability (CIN) and microsatellite instability (MSI), mainly induces tumorigenesis by activating proto-oncogenes. The MTHFR gene is a common original gene with two common forms. Among them, C677T and A1298C interfere with normal folate metabolism, thereby increasing the risk of CRC [58]. Studies indicate that the C677T variant is associated with reduced CRC risk; when folate intake is sufficient, cancer risk in carriers may decrease. However, insufficient folate intake may impair DNA methylation and synthesis/repair, elevating CRC risk. Further animal studies show that folate deficiency causes exon-specific hypomethylation of the p53 gene and increased DNA methyltransferase activity, though moderate folate deficiency does not necessarily alter DNA methylation [59]. Additionally, the HER3 gene exhibits upregulation and hypomethylation in CRC cases. Highly expressed and hypomethylated HER3 may play a significant role in the early stages of CRC tumorigenesis [60].

### 4.2. Histone Modification

Multiple covalent modifications on the N-terminal tails of histones cooperatively regulate chromatin configuration and gene expression. In CRC, the histone modification profile is significantly altered, primarily due to abnormal expression or mutations of histone-modifying/demodifying enzymes. Histone acetylation is typically associated with transcriptional activity, catalyzed by HATs and removed by HDACs [61]. Studies show reduced global histone acetylation levels in CRC tumor tissues compared to normal tissue. One study found that H3K9, H3K27, and H3K56 acetylation were significantly lower in tumors of CRC patients than in adjacent tissues [62]. Further research revealed that metabolic enzymes responsible for acetyl-CoA production, such as ACSS2, are downregulated in CRC, leading to insufficient donor supply for histone acetylation. Additionally, multiple HDACs are overexpressed in CRC, promoting deacetylation-mediated silencing of tumor suppressor genes. High expression of HDAC1 and HDAC2 correlates with CRC stage progression, while Sirtuin family members SIRT1 and SIRT2 are often considered oncogenes; their hyperactivation in CRC promotes cell survival. Research by Guan et al. found that HDAC2 overexpression in CRC epigenetically silences NLRP3 by removing H3K27 acetylation from its promoter, reducing chromatin accessibility and blocking recruitment of the BRD4-phosphorylated P65 complex [63]. This suppresses GSDMD-mediated pyroptosis and diminishes chemotherapy efficacy. Similarly, Yao et al. demonstrated that PRMT1 directly recruits the SWI/SNF complex ATPase subunit SMARCA4 by catalyzing asymmetric dimethylation of histone H4 arginine 3 (H4R3me2a) [64]. This recruitment localizes SMARCA4 to promoters of key proliferation and migration genes like EGFR and TNS4, synergistically enhancing their transcriptional activity, activating the EGFR signaling pathway, and thereby promoting proliferation and migration in CRC cells. Conversely, SIRT6 is frequently lost or under-expressed in CRC. Acting as a corepressor of HIF-1α and Myc, its loss relieves inhibition of glycolysis and glutamine metabolism, promoting tumor growth. Common alterations in CRC include imbalances in histone methyltransferase and demethylase expression. Overexpression of EZH2 (which mediates H3K27 trimethylation) in CRC is associated with poor prognosis. Abnormalities in histone demethylases like KDM5/6 can also disrupt transcriptional regulation. JMJD2B (KDM4B), an H3K9me3 demethylase, is induced by HIF1 in the hypoxic CRC environment. Its interaction with HIF1α removes H3K9 trimethyl marks, activating multiple glycolysis-related and angiogenesis genes to promote cancer cell proliferation and invasion. Furthermore, research by Liu et al. found that SNORA28 acts as a molecular decoy to recruit BRD4, enriching it at the LIFR promoter and elevating H3K9 acetylation levels in this region, thereby enhancing LIFR transcriptional activity [65]. Subsequently, JAK1/STAT3 signaling is persistently activated, driving CRC cell proliferation and reducing radiosensitivity. Intriguingly, Yang et al. discovered that CRC cells exhibit intrinsic tolerance to ferroptosis, which histone deacetylase inhibitors (HDACi) can significantly disrupt [66]. HDACi specifically inhibit HDAC1 and reduce its K412 lactylation level, thereby increasing H3K27 acetylation at the FTO and ALKBH5 promoters to activate these two m^6A demethylases. Enhanced activity of FTO and ALKBH5 removes m^6A modifications on FSP1 mRNA, leading to accelerated FSP1 mRNA degradation and suppressed expression of this ferroptosis-inhibiting protein. FSP1 downregulation impairs the cell’s ability to clear lipid peroxides, rendering CRC cells highly sensitive to ferroptosis inducers.

### 4.3. Noncoding RNA Dysregulation

In the human body, the vast majority of RNA is ncRNA, which regulates gene expression and stabilizes normal life activities. The expression profiles of noncoding RNAs are widely dysregulated and participate in metabolic and epigenetic regulatory circuits [67] (Table 1).

#### 4.3.1. miRNA

miRNA is a typical type of small molecule RNA that regulates gene expression by binding to the 3′ untranslated region of target mRNA. In CRC, miR-34 and miR-143/145 are downregulated or lost, leading to upregulation of corresponding pro-oncogenic genes [68,69,70]. Conversely, oncogenic miRNAs like miR-21 and miR-135b are overexpressed, promoting tumorigenesis. Notably, studies indicate that miRNA networks participate in regulating tumor metabolism and signaling pathways. For instance, miR-1 and miR-30a-5p reduce HIF-1α expression by targeting its mRNA, indirectly downregulating glycolytic genes such as GLUT1 and HK2, thereby suppressing glycolysis and proliferation in CRC cells [71,72]. Conversely, miR-135b overexpression in CRC targets and inhibits upstream factors of the AMP-activated protein kinase (AMPK) pathway, impairing cellular energy sensing and shifting metabolism toward biosynthesis, which is closely linked to increased CRC invasiveness [73].

#### 4.3.2. LncRNAs

LncRNAs are extensively dysregulated in CRC. LncRNAs exceed 200 nt in length and regulate gene expression and cellular functions through diverse mechanisms. Specifically expressed in CRC, lncRNA CCAT1-L originates upstream of the MYC gene and mediates long-range chromatin looping between the MYC promoter and enhancer, thereby enhancing MYC transcription [74]. Research shows that lncRNA GLCC1 is upregulated in CRC; it stabilizes c-Myc protein by binding to HSP90, preventing c-Myc from ubiquitin-mediated degradation and promoting c-Myc-dependent glucose metabolism and tumor progression [75]. Another lncRNA LINRIS, binds to IGF2BP2 protein to block its autophagic degradation, enhancing IGF2BP2-mediated stabilization of c-Myc target gene mRNAs (e.g., GLUT1, PKM2) [76]. This increases glycolytic flux and promotes CRC cell proliferation. These findings demonstrate that lncRNAs can epigenetically influence transcription factors or mRNA stability, thereby remodeling metabolic pathways.

#### 4.3.3. CircRNAs

CircRNAs, a class of circular RNAs formed by back-splicing of precursor mRNAs, have recently been identified as functionally significant in CRC. CRC adapts to nutritional and stress contexts through a circRNA network that coordinately rewires metabolism, inflammatory signaling, and cell adhesion, thereby sustaining growth and acquiring metastatic competence. circINSIG1 is upregulated and translated into a microprotein, circINSIG1-121, which functions as a scaffold to recruit the CUL5–ASB6 E3 ubiquitin ligase complex to INSIG1, promoting K48-linked ubiquitination and degradation, relieving suppression of the SCAP–SREBP2 pathway, elevating cholesterol biosynthesis, and supporting proliferation and distant dissemination [77]. By contrast, circPLCE1 encodes a small peptide that drives ubiquitin-dependent degradation of RPS3, a noncanonical cofactor of NF-κB; the resulting reduction in NF-κB transcriptional activity suppresses tumor growth and metastasis, indicating that coding circRNAs can also down-modulate proinflammatory oncogenic signaling [78]. Under nutrient stress, cytoplasm-enriched hsa_circ_0062682 acts as a competing endogenous RNA that sequesters miR-940, de-repressing the rate-limiting serine-biosynthesis enzyme PHGDH, increasing serine flux and the supply of NADPH and glutathione, lowering oxidative stress, and promoting proliferation [79]. During metastatic progression, circHERC4 sponges miR-556-5p to upregulate the transcriptional corepressor CTBP2, which in turn represses E-cadherin, weakening intercellular adhesion and enhancing migration and invasion [80].

Furthermore, circACC1 is induced under metabolic stress; it acts as a molecular scaffold by binding to AMPKβ1 and γ1 subunits, promoting assembly and stabilization of the AMPK holoenzyme complex, thereby enhancing AMPK activity [81]. Activated AMPK inhibits acetyl-CoA carboxylase (ACC1) to reduce fatty acid synthesis while modulating 6-phosphofructo-2-kinase (PFK2) activity to influence glycolysis. Through this mechanism, circACC1 links energy-sensing signals to metabolic pathway regulation, contributing to metabolic adaptation in CRC. Similarly, circRNA MYH9 binds to hnRNPA2B1, altering the splicing of p53 pre-mRNA [82]. This reduces functional p53 protein levels, relieving its transcriptional repression on serine and glycine synthesis pathways, thereby enabling cancer cells to acquire more one-carbon units for biosynthesis.

Collectively, these studies delineate a continuous circuitry in which coding and ceRNA-type circRNAs jointly regulate cholesterol and one-carbon metabolism, inflammatory transcriptional programs, and adhesion phenotypes, providing a clear mechanistic rationale for combination interventions targeting circRNAs themselves, their translated microproteins, or downstream enzymatic and adhesion nodes.

## 5. Interplay Between Metabolism and Epigenetic Modifications in CRC Development

Metabolic reprogramming and epigenetic modifications do not operate independently during CRC progression. Instead, they interact and couple through multilayered mechanisms. Changes in metabolic status can transmit intracellular environmental information to the nucleus by altering the quantity and types of metabolites, thereby regulating epigenetic enzyme activity and chromatin states [83]. Conversely, epigenetic mechanisms can feedback to influence cellular metabolic programs by controlling the expression of metabolism-related genes and the activity of metabolic enzymes. This bidirectional “metabolism-epigenetics” crosstalk plays a crucial role in signal transduction, gene expression, and tumor microenvironment adaptation. Below, we discuss two aspects: metabolic regulation of epigenetics and epigenetic regulation of metabolism.

### 5.1. Metabolic Regulation of Epigenetics

Tumor metabolic reprogramming often alters the abundance of key metabolites, which directly impact epigenetic modification processes by determining enzyme activity and modification levels. Notable examples include.

#### Metabolic Substrates Influence Epigenetic Enzyme Activity

Tumor metabolic reprogramming often alters the levels of key metabolites, which can directly impact epigenetic modification processes by determining enzyme activity and modification levels.

(1) SAM

S-Adenosylmethionine (SAM), generated by the methionine cycle, serves as the primary methyl donor in cells and participates in DNA and histone methylation reactions. SAM produces S-adenosylhomocysteine (SAH) upon methyl transfer, with SAH acting as a potent inhibitor of methyltransferases [84]. The intracellular SAM/SAH ratio is a critical determinant of DNMT and histone methyltransferase (HMT) activity. In CRC, metabolic changes can elevate SAM levels, thereby increasing methylation levels and DNMT activity. Treating CRC cells with SAM downregulates EMT-related genes like TGFB1 through promoter methylation at specific sites—without significantly altering global genome-wide methylation—leading to S-phase arrest and induction of a senescent phenotype. Concurrently, increased γ-H2AX indicates activation of the DNA damage response, with elevated expression of repair genes such as HUS1. SAM primarily inhibits CRC progression by inducing cellular senescence and enhancing DNA repair [85]. However, its benefit to genomic stability depends on the tumor’s unique molecular characteristics. Furthermore, research by Zhang et al. demonstrated that PHGDH undergoes monoubiquitination at Lys146 in CRC cells via the Cullin4A-E3 ligase complex. This modification recruits the chaperone DNAJA1, promoting PHGDH tetramer formation and enhancing enzymatic activity, thereby increasing serine-glycine synthesis and SAM levels [86]. Elevated SAM activates transcription of cell adhesion genes such as LAMC2 and CYR61 through SET1A-mediated H3K4 trimethylation, enhancing cancer cell migration and driving CRC metastasis. Exogenous SAM supplementation increases the SAM/SAH ratio in CRC cells, activating DNMT and HAT activity and significantly upregulating DNA and histone methylation levels. Conversely, endogenous SAM deficiency caused by dietary or metabolic defects (e.g., folate/methionine deficiency reducing methyl donors) triggers genome-wide hypomethylation and unstable gene expression. One study confirmed that adjusting dietary methyl donor intake (e.g., methionine and folate) influences DNA methylation status in CRC tissues. Additionally, cancer stem cells exhibit extremely high demand for methyl donors; inhibiting methionine adenosyltransferase MAT2A depletes SAM, impairs histone methylation, and suppresses cancer stem cell self-renewal.

(2) Acetyl-CoA

Acetyl-CoA is a key intermediate connecting glucose, lipid, and amino acid metabolism, and the sole acetyl group donor for histone acetylation. Cellular acetyl-CoA levels are regulated by nutritional status: feeding states promote glycolysis and fatty acid oxidation to generate abundant acetyl-CoA [87], while fasting states reduce it. These fluctuations cause global protein acetylation changes. Tumor metabolic reprogramming often enhances acetyl-CoA production. In CRC, not only can conventional carbon sources like glucose be converted to citrate and then acetyl-CoA, but acetate can also serve as an alternative carbon source for acetyl-CoA generation. One study found that acetate supplementation in CRC cells significantly elevates nuclear acetyl-CoA levels and increases acetylation of histones H3K9, H3K27, and H3K56 [88]. Similarly, Butyrate produced by the fermentation of dietary fiber by the gut microbiota can enter intestinal epithelial cells, raising Ac-CoA levels and enhancing histone acetylation. Butyrate treatment increases histone acetylation at promoters of DNA mismatch repair genes in CRC cells, promoting their expression. When acetyl-CoA production is limited, histone acetylation decreases, impairing normal gene expression. In CRC, downregulation of ACSS2 enzyme reduces acetate utilization, leading to acetyl-CoA deficiency. This process lowers histone acetylation levels, ultimately suppressing tumor suppressor gene expression [89]. Additionally, ACLY converts mitochondrial citrate to cytosolic acetyl-CoA and is a primary source of histone acetylation under normal growth conditions. Silencing ACLY in CRC cells significantly reduces global histone acetylation.

(3) TCA metabolites and demethylases

TCA cycle metabolites, such as α-ketoglutarate, succinate, and fumarate—also regulate epigenetic enzyme activity. Both DNA demethylase TET and JmjC-family histone demethylases require α-KG and Fe^2+^ as cofactors for catalytic function [90]. Thus, reduced α-KG levels or accumulation of its analogs significantly inhibit these demethylases. Although IDH gene mutations are rare in CRC, in other cancers, IDH mutations convert α-KG to 2-hydroxyglutarate (2-HG). Structurally similar to α-KG, 2-HG competitively occupies the α-KG binding site of these enzymes, inhibiting TET and JMJD activity and blocking DNA/histone demethylation. In IDH1-mutant CRC cells, 2-HG accumulation decreases 5hmC, induces DNA hypermethylation, and promotes malignant phenotypes [91]. KRAS-mutant CRC cells often exhibit succinate and fumarate accumulation with reduced α-KG due to altered mitochondrial metabolism. Succinate and fumarate act as α-KG antagonists, similarly inhibiting TET and JMJD activity. Studies confirm that excess succinate increases DNA methylation at key gene promoters by blocking TET activity, enhancing Wnt/β-catenin signaling in KRAS-mutant CRC [92].

(4) NAD^+^/NADH and deacetylases

NAD^+^ is an essential cofactor for Sirtuin deacetylases. Sirtuins act as metabolic sensors, consuming NAD^+^ to perform deacetylation. The cellular NAD^+^/NADH ratio directly impacts Sirtuin activity and downstream epigenetic effects [93]. In CRC, NAD^+^ synthase NAMPT is significantly upregulated, increasing cellular NAD^+^ levels and the NAD^+^/NADH ratio. Further research shows that c-Myc transcriptionally activates NAMPT to elevate NAD^+^ levels, enhancing SIRT1 activity. SIRT1-mediated deacetylation then influences metabolism and gene expression, promoting cancer cell proliferation and survival [94]. Concurrently, Myc also enhances LDHA activity, reducing pyruvate to lactate while oxidizing NADH to NAD^+^, thereby maintaining high NAD^+^ levels for sustained SIRT1 activation.

Nicotinamide N-methyltransferase (NNMT) has emerged as a central node linking metabolic reprogramming to epigenetic remodeling in colorectal cancer [95]. NNMT transfers a methyl group from S-adenosylmethionine (SAM) to nicotinamide to generate 1-methylnicotinamide and S-adenosylhomocysteine (SAH), which lowers the intracellular SAM/SAH ratio and creates a methyl-sink effect that diminishes the availability of methyl donors for DNA and histone methyltransferases. The resulting global hypomethylation and redistribution of chromatin marks reshape transcriptional programs that favor tumor progression and therapy resistance [96]. In CRC, NNMT is frequently upregulated and associates with adverse clinicopathologic features, underscoring its biological and translational relevance. Beyond methyl-donor depletion, NNMT perturbs nicotinamide and NAD pools and intersects with sirtuin-dependent deacetylation, adding a second regulatory axis through which it can influence metabolic gene expression and inflammatory signaling [97]. A growing portfolio of NNMT inhibitors, including substrate-competitive and bisubstrate-mimetic chemotypes, demonstrates nanomolar enzymatic potency and on-target activity in preclinical models, with early evidence for anti-tumor and chemosensitizing effects that could be exploited in CRC treatment. Together, these data nominate NNMT as both a biomarker of metabolic–epigenetic state and a druggable vulnerability, and they argue for biomarker-guided trials that incorporate pharmacodynamic readouts of SAM/SAH balance, DNA and histone methylation, and NAD–sirtuin pathway activity to validate mechanism and clinical utility in CRC [98,99,100].

### 5.2. Epigenetic Regulation of Metabolism

Epigenetic modifications can directly shape cellular metabolic profiles by regulating processes such as transcription of metabolism-related genes. In CRC, aberrant promoter methylation status or chromatin states of numerous metabolic enzyme and transporter genes lead to dysregulated expression, thereby altering metabolic pathway activity. Studies show that METTL3 enhances REG1α transcript stability via m^6A modification, significantly elevating its levels in CRC tissues and serum. Highly expressed REG1α activates β-catenin signaling, further upregulating transcription factor MYC. MYC subsequently promotes expression of glycolytic genes like LDHA, markedly increasing lactate production and glucose uptake, thereby accelerating tumor cell proliferation and invasion [20]. Research by Bai et al. revealed that in CRC, CENP-N directly binds to SEPT9 and induces methylation at specific lysine sites. This modification enhances SEPT9 activity, subsequently upregulating key glycolytic enzymes such as GLUT1, HK2, and PKM2. Further investigation demonstrated that this process drives CRC cells to shift metabolism toward aerobic glycolysis, providing sufficient energy and intermediate metabolites to support migration and metastasis. In mouse orthotopic and liver metastasis models, tumors overexpressing CENP-N formed faster and developed more metastatic foci, whereas silencing CENP-N significantly suppressed glycolysis and attenuated liver metastasis [101]. Additionally, beyond methylation modifications, other histone modifications also regulate metabolic pathways. Research has found that PHGDH undergoes monoubiquitination at Lys146 under the action of the Cullin4A-E3 ligase complex. This ubiquitin modification recruits the chaperone protein DNAJA1, promoting PHGDH tetramer formation and enhancing its enzymatic activity. Consequently, serine-glycine synthesis and SAM levels increase. Elevated SAM activates transcription of cell adhesion genes such as LAMC2 and CYR61 through SET1A-mediated H3K4 trimethylation, enhancing CRC cell migration and driving CRC metastasis [86,102]. Bevacizumab inhibits angiogenesis, creating tumor hypoxia. This hypoxic state triggers enhanced glycolysis in cancer cells, leading to lactate accumulation in the TME. Lactate is utilized as a substrate for lactylation modification at lysine 18 of histone H3 (H3K18la). This epigenetic alteration upregulates transcription of the autophagy regulator gene RUBCNL (Pacer). The Pacer protein binds to Beclin-1, promoting recruitment of the class III PI3K complex, driving autophagosome maturation and sustaining the proliferation and survival of hypoxic cancer cells. This process ultimately diminishes the efficacy of bevacizumab. Inhibiting histone lactylation in mouse models significantly suppresses tumor growth and restores sensitivity to bevacizumab [103]. In CRC, histone acetyltransferase KAT2A increases H3K27 acetylation levels at the PTCD3 promoter, thereby enhancing PTCD3 transcription. Elevated PTCD3 forms a complex with the mRNA stability factor IGF2BP2, assisting its binding to and stabilization of SLC38A2 mRNA (encoding the glutamine transporter). This results in massive expression of SLC38A2 protein. SLC382 imports more glutamine into cells, providing substrate for GLS1-driven glutaminolysis. This significantly boosts the energy and carbon skeleton supply required by CRC cells, accelerating their proliferation, migration, and invasion [104]. Certain ncRNAs also regulate metabolism. Under hypoxia, CRC cells significantly upregulate a newly identified circular RNA: circINSIG1. circINSIG1 is not only highly expressed in tumor tissues and associated with advanced stages and poor survival but also possesses coding potential, translating into a 121-amino acid peptide, circINSIG1-121. This peptide binds to the E3 ligase complex CUL5-ASB6, recruiting it to INSIG1 (an inhibitor of cholesterol metabolism). This promotes K48-linked ubiquitination of INSIG1 at Lys156 and Lys158, leading to its degradation. Further research revealed that this enhances the cholesterol biosynthesis pathway, increases lipid synthesis in CRC cells, and significantly elevates their proliferation and metastatic capabilities [77].

In CRC, metabolic control of epigenetic enzymes and epigenetic control of metabolic programs form a bidirectional loop. Divergent reports on acetyl-CoA availability and acetate or butyrate effects on histone acetylation are largely explained by substrate accessibility and spatial compartmentalization: local carbon inputs can elevate acetylation at selected loci, whereas global acetyl-CoA scarcity reduces overall acetylation levels. Lactate accumulation and hypoxia can selectively increase histone lactylation and reshape autophagy or mismatch-repair programs, although many observations come from short-term treatments and in vitro labeling, and long-timescale causal chains in tumors remain to be defined. For DNA and histone methylation, mechanistic coupling among the SAM to SAH balance, PHGDH-driven one-carbon flux, and KAT or HDAC activity is clear in models, yet human studies often lack harmonized pre-analytics and independent external cohorts, which contributes to inconsistent methylation trends at the same loci. Future work should obtain longitudinal paired tissue and liquid biopsies within the same patients, jointly measuring metabolic-flux surrogates and epigenetic pharmacodynamics under standardized quality control and reporting thresholds to improve cross-platform reproducibility.

## 6. Crosstalk Between Epigenetic Modifications, Metabolic Reprogramming, and the TIME

**Figure 3:**
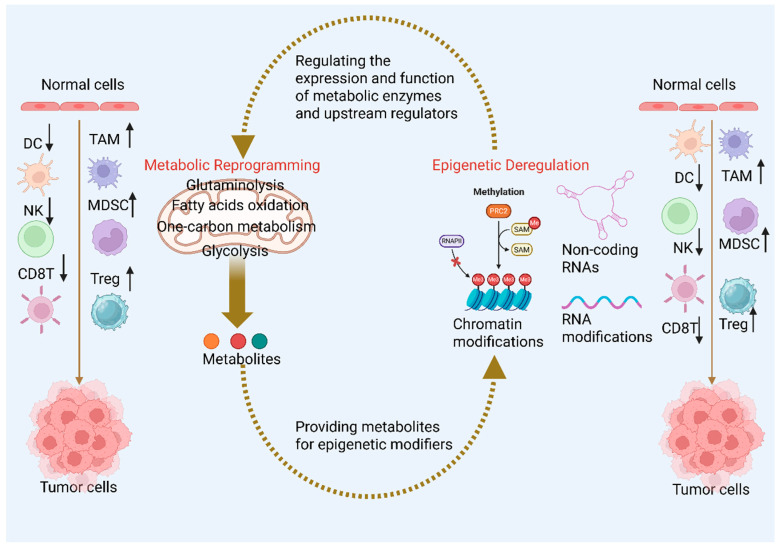
The interaction mechanism between metabolic reprogramming and epigenetic modification in CRC’s TIME. Tumor cells induce the formation of an immunosuppressive microenvironment by regulating metabolic reprogramming and epigenetic modifications. Metabolic reprogramming and epigenetic modifications interact to form a positive feedback regulatory mechanism, ultimately promoting immune escape by tumor cells. The figure was created with BioRender.com.

The interplay between metabolism and epigenetics in CRC significantly impacts the tumor immune microenvironment (TIME). Metabolic reprogramming products such as lactate and keto acids not only affect tumor cell epigenetics but also diffuse or signal to influence the epigenetic states and functions of nearby immune cells [105] (Figure 3).

### 6.1. T Cell

For T cells, metabolic reprogramming and epigenetic modifications markedly suppress the anti-tumor function of CD8^+^ T cells while increasing Treg cell infiltration. Research by Wang et al. revealed that glycerol-3-phosphate acyltransferase 3 (GPAT3) is significantly upregulated in CRC cells. Acetylation at its K316 site enhances catalytic activity for synthesizing lysophosphatidic acid, driving massive lipid droplet accumulation. Enriched lipid droplets impair oxaliplatin-induced immunogenic cell death, reduce cytotoxic factor release (e.g., IFN-γ), and promote CD8^+^ T cell exhaustion in the TIME, thereby enhancing tumor survival and driving liver metastasis and chemotherapy resistance [106]. Zi and colleagues found that the metabolic hub gene SIRT1 is significantly upregulated in CRC cells, promoting CX3CL1 secretion by enhancing glucose-lipid conversion. CX3CL1 binds to CX3CR1 on Treg cells in the TIME, activating transcription factors SATB1 and BTG2 to drive the differentiation of TCF7^+^ precursor Tregs into TNFRSF9^+^ highly functional Tregs. Thus, tumor metabolic reprogramming and enhanced CX3CL1-CX3CR1 signaling synergistically increase Treg infiltration and immunosuppression, fueling CRC progression. CX3CR1 inhibitors (alone or combined with PD-1 antibodies) reverse this immunosuppressive effect and improve immunotherapy efficacy in mouse models [107].

### 6.2. MDSCs

MDSCs are an immunosuppressive immune cell population in the TIME. Du et al. discovered that loss of the histone demethylase UTX in CRC cells confers methylation-mediated protection to phenylalanine hydroxylase (PAH), preventing its degradation and sustaining its activity [108]. This leads to massive intracellular tyrosine synthesis and secretion into the TIME. Tumor-infiltrating MDSCs uptake tyrosine, which is metabolized by 4-hydroxyphenylpyruvate dioxygenase (HPPD) into homogentisic acid. Homogentisic acid induces carbonylation of PIAS3 (a STAT3 inhibitor) at Cys176, relieving PIAS3-mediated suppression of STAT5. This enhances STAT5-dependent survival and expansion signals, promoting MDSC accumulation and immunosuppression in tumors. Additionally, another study found that the intestinal fungus Candida tropicalis triggers Syk kinase activation via its cell wall sugar-binding receptor Dectin-3. Activated Syk binds to and phosphorylates pyruvate kinase M2 (PKM2) at Tyr105. Phosphorylated PKM2 translocates to the nucleus of MDSCs, forming a complex with HIF-1α to upregulate transcription of glycolytic enzymes (e.g., GLUT1, PKM2, LDHA). This dramatically enhances aerobic glycolysis in MDSCs. Reprogrammed MDSCs secrete more NO and ROS, accompanied by elevated COX2 and NOX2 expression, intensifying immunosuppression to suppress CD8^+^ T cell function and drive CRC progression [109].

### 6.3. M2 Macrophages

For macrophages, these alterations inhibit M1 macrophage function while promoting M2 macrophage proliferation and activity. For macrophages, these crosstalk mechanisms inhibit M1 macrophage function while promoting M2 macrophage proliferation and activity. Research by Liu et al. revealed that lactate secreted by CRC cells upregulates VSIG4 expression by altering epigenetic modifications at the VSIG4 gene promoter in macrophages. High VSIG4 levels activate JAK2/STAT3 signaling, enhancing fatty acid oxidation and inducing PPAR-γ upregulation, thereby driving TAMs toward an M2 phenotype. M2 macrophages subsequently release large amounts of heparin-bound EGF, promoting cancer cell proliferation, invasion, and immune evasion [110]. Under chemotherapy stimulation, interactions between CRC cells and TAMs trigger massive CXCL7 secretion from TAMs. CXCL7 binding to CXCR2 receptors on tumor cells activates the STAT1-dependent interferon pathway, upregulating PHGDH and enhancing serine synthesis. This increases the production and secretion of SAM. Exported SAM reciprocally polarizes macrophages toward an immunosuppressive M2 phenotype while sustaining high CXCL7 expression, forming a CXCL7-SAM positive feedback loop that remodels the tumor microenvironment and confers chemotherapy resistance to CRC cells [111]. In the CRC microenvironment, TAMs aberrantly express the lipolytic coactivator ABHD5. ABHD5 suppresses ROS-mediated expression of the transcription factor C/EBPɛ, blocking SRM gene transcription and reducing spermine synthesis in macrophages. Spermine deficiency impairs the tumor-suppressive capacity of M1 macrophages, thereby promoting tumor cell growth [112]. Intriguingly, Zhang et al. demonstrated that silencing Agpat4 in CRC tissues causes tumor cells to release more lysophosphatidic acid (LPA). Acting through LPAR1/3 on TAMs, LPA polarizes them into an M1-like phenotype characterized by high p38-p65 activation and proinflammatory cytokine secretion. Inflammatory factors produced by M1 macrophages further promote CD4^+^ and CD8^+^ T cell infiltration and activation, thereby enhancing anti-tumor immunity and suppressing cancer progression [113].

### 6.4. Other Immune Cells

In the TIME, beyond the common immune cells mentioned above, DCs and NK cells also undergo a series of changes that further induce immunosuppression. In CRC, elevated expression of the RNA-binding protein MSI2 directly binds to the 3′-UTR of HMGB1 mRNA (nucleotides 1403–1409), enhancing its translation efficiency and favoring the generation of the oxidized disulfide bond conformation. Simultaneously, MSI2 interacts with the cytoplasmic acetyltransferase P300, increasing P300 levels and promoting acetylation of HMGB1 at lysine 29. This coordinated translation and acetylation drive HMGB1 translocation from the nucleus to the cytoplasm and its massive secretion into the extracellular space. Released HMGB1 acts as a DAMP molecule, stimulating dendritic cell maturation and migration, inducing CD8^+^ T cell infiltration and exacerbated inflammation, ultimately promoting the pathological progression of CRC [114]. For NK cells, both infiltration and function are significantly suppressed in the TME. Research by Han and colleagues revealed that overexpression of the long noncoding RNA ELFN1-AS1 in CRC cells enhances the binding of the acetyltransferase GCN5 to the transcriptional coregulator SND1 by acting as a scaffold. This elevates H3K9 acetylation levels at the GDF15 promoter region, leading to massive GDF15 transcription. Secreted GDF15 activates JNK signaling, downregulating the NK cell-killing essential receptor NKG2D and the effector molecule granzyme B (GZMB), thereby impairing NK cell cytotoxicity and enabling tumor cells to evade immune surveillance [115].

## 7. Metabolic Plasticity of Cancer Stem Cells and Therapeutic Resistance

Cancer stem cells systematically reprogram central carbon, amino-acid, redox, and lipid metabolism to sustain self-renewal, adapt to metabolic stress, and evade cytotoxic injury; they therefore serve as metabolic hubs in acquired resistance and distant metastasis.

In CRC stem cells, lactate supply increases p300-mediated histone lactylation at H4K12, which augments transcription of the glutathione-synthesis gatekeeper GCLC, raises cellular reducing capacity, suppresses lipid peroxidation, and ultimately lowers the incidence of ferroptosis. Inhibition of p300 or LDHA decreases this epigenetic mark and GCLC expression and enhances oxaliplatin cytotoxicity, whereas direct GCLC blockade restores ferroptotic susceptibility [116]. In parallel, SOX2 functions as an amplifier that couples metabolism and signaling in colorectal cancer: it transcriptionally activates the efflux pump ABCC2 and increases the nuclear localization and transcriptional activity of β-catenin, while upregulating Beclin1 to drive autophagic flux [117]. These two arms cooperatively consolidate chemoresistance, spheroid formation, and epithelial–mesenchymal transition. At the level of mitochondrial one-carbon metabolism, MTHFD2 is a key enzyme for purine synthesis that preserves stem-like features and contributes to resistance to EGFR tyrosine kinase inhibitors such as gefitinib. Genetic or pharmacological inhibition of MTHFD2, or restriction of purine supply, functionally restores TKI sensitivity and attenuates stemness [118]. With respect to energy and redox coupling, the adenylate kinase hCINAP promotes phosphorylation of LDHA at Y10, thereby increasing glycolytic flux and reactive oxygen species buffering, which supports self-renewal and invasiveness of colorectal cancer stem cells [119]. hCINAP inhibition markedly reduces spheroid formation and tumor initiation in orthotopic or xenograft models, indicating a proximal switch that links energy metabolism to stemness maintenance. Lipid metabolism also directly governs stemness: the lncRNA ROPM stabilizes PLA2G16 mRNA, increases phospholipase activity and the supply of free fatty acids, particularly arachidonic acid, and subsequently activates PI3K/AKT, Wnt/β-catenin, and Hippo/YAP pathways to sustain stemness and enhance chemoresistance [120]. Along this axis, combining PLA2 inhibition with conventional chemotherapy facilitates depletion of stem-like populations.

Nutritional interventions can serve as auxiliary levers for metabolic sensitization. Methionine restriction upregulates miR-320d and represses its target c-Myc, thereby lowering expression of efflux mediators such as ABCG2 and increasing the sensitivity of colorectal cancer stem cells to 5-fluorouracil; this sensitization is blunted when miR-320d is inhibited or c-Myc is restored [121]. Microenvironmental cues likewise remodel stem-cell metabolism and phenotype. Exosomes derived from human umbilical-cord mesenchymal stem cells are enriched for miR-486-5p, which directly targets NEK2, reduces glycolytic throughput, and weakens stemness features in colorectal cancer cells [122]. NEK2 reconstitution reverses these changes, indicating an intervention-ready pathway from niche signals to metabolism and stemness.

Taken together, cancer stem cell metabolism is tightly linked to therapeutic resistance. To enhance clinical implementability, future studies should prioritize biomarker-guided stratified trials, use pre–post pharmacodynamic readouts, and validate real-world effectiveness and safety across multiple centers. The goal is to establish reproducible and mechanistically consistent combinations that integrate metabolic targeting with standard anticancer therapies.

## 8. Novel Directions in Diagnosis

Metabolic and epigenetic abnormalities in tumors provide rich biomarkers and novel technological opportunities for CRC diagnosis. Recent advances in multi-omics integration, single-cell sequencing, and liquid biopsy offer promise for improving early screening and personalized diagnosis of CRC (Table 2).

### 8.1. Early Detection

Traditional diagnosis often relies on single-dimensional molecular markers, whereas CRC development results from multi-layered changes. Integrating multi-omics data can comprehensively reveal molecular features of CRC pathogenesis and identify specific diagnostic biomarkers. Large-scale projects like TCGA, through whole-genome sequencing, DNA methylation profiling, mRNA/miRNA expression analysis, and proteomics of CRC, proposed the Consensus Molecular Subtyping (CMS), classifying CRC into four subtypes. These include CMS3 (characterized by metabolic dysregulation) and CMS1 (enriched for high immune infiltration/hypermethylation) [123]. This subtyping, integrating metabolic and epigenetic features, aids in prognosis assessment and treatment strategy selection. Multi-omics approaches also show potential in early CRC screening. The multi-target stool FIT-DNA test (e.g., Cologuard) is clinically used, combining fecal occult blood testing with stool DNA analysis for mutations (e.g., KRAS) and methylation markers (e.g., NDRG4, BMP3 promoter methylation), improving screening sensitivity. Future multi-omics machine learning models may integrate patient data such as gene mutations, cell-free DNA methylation, plasma metabolite profiles, and gut microbiota for risk assessment. Current research explores combining indicators—e.g., simultaneously analyzing methylation profiles, mutations, fragmentation patterns of ctDNA in blood, and circulating protein biomarkers—to build multi-factor discriminant models for early CRC detection. Research by Mo et al. identified multiple plasma cfDNA methylation biomarkers (e.g., BCAN, BCAT1, IKZF1, Septin9_1, Septin9_2, VAV3) for CRC detection. Patients positive for these six targets had a 17-fold higher recurrence risk than negative patients [124].

### 8.2. Diagnostic Approaches

#### 8.2.1. Single-Cell and Spatial Omics Analysis

Given high tumor heterogeneity, scRNA-seq and spatial transcriptomics dissect fine-grained features of tumors and their microenvironments. scRNA-seq identifies distinct cell populations and their metabolic/epigenetic states at single-cell resolution. One study analyzing CRC and adjacent tissues identified eight major cell types and 25 subpopulations, revealing significant metabolic and immune phenotypic heterogeneity among subpopulations [125]. However, terminally differentiated CD8^+^ T cells, myeloid cells, and fibroblasts converged toward high lipid metabolism and immunosuppressive functions, governed by shared transcription factors. This suggests that diverse cells in the TME evolve toward similar metabolic-immune states, with lipid remodeling as a key trigger for immunosuppression. Immune and clinical risk models built from single-cell data show excellent prognostic value. Spatial transcriptomics maps gene expression to tissue architecture, linking histology with molecular features. Combined with multiplex immunofluorescence, it revealed metabolically inactive tumor regions enriched in SFRP2^+^ CAFs and Tregs, forming immunosuppressive niches resistant to chemotherapy [126]. Clinically, single-cell and spatial omics may enable “fingerprint” profiling of tumor metabolic-epigenetic features for personalized diagnosis and treatment.

#### 8.2.2. Liquid Biopsy and Non-Invasive Testing

Liquid biopsy non-invasively monitors tumor dynamics via tumor-derived components in blood or body fluids. For CRC, plasma ctDNA, circulating tumor cells (CTCs), exosomes, and platelets are active research areas. ctDNA methylation markers demonstrate high specificity for early CRC detection [127]. As reviewed, cfDNA detection of aberrantly methylated genes (e.g., SDC2, TFPI2, NDRG4) shows promise in early screening. Current applications include ctDNA methylation-based tests (e.g., Septin9 methylation) and multi-gene methylation panels for screening and adjuvant monitoring. CTC analysis provides whole-genome and phenotypic tumor cell information. Advanced single-cell CTC sequencing can resolve metabolic gene expression and epigenetic states to predict metastatic potential [128]. Exosomal miRNAs and metabolites are also potential non-invasive markers, e.g., elevated exosomal metabolic enzyme mRNAs or miRNAs (e.g., miR-21) in CRC patient plasma correlate with disease progression [129]. Future multi-omics liquid biopsies will simultaneously analyze ctDNA genomics/epigenomics, exosomal RNAs, circulating proteins, and metabolites in one blood sample, using AI algorithms for integrated assessment [130]. This approach may improve sensitivity, provide comprehensive molecular tumor profiles, and even detect minimal residual disease (MRD). In summary, liquid biopsy combined with metabolic and epigenetic indicators offers novel tools for early diagnosis, treatment efficacy evaluation, and recurrence monitoring in CRC. Its non-invasive and repeatable nature will significantly advance personalized medicine.

#### 8.2.3. Multi-Omics Integrative Analysis

Single-dimensional biomarkers often fail to capture CRC heterogeneity. Multi-omics integration enables precise diagnosis by combining genomic, epigenetic, transcriptomic, proteomic, metabolomic, and microbiomic data to identify CRC-specific molecular features and subtypes. TCGA-classified CMS subtypes (e.g., metabolically dysregulated CMS3 and hyperimmune/hypermethylated CMS1) correlate with prognosis and treatment response, demonstrating diagnostic value [131]. Novel stool tests like Cologuard combine stool DNA methylation (e.g., NDRG4, BMP3 promoter methylation) and gene mutations to enhance sensitivity. Blood-based ctDNA methylation markers (e.g., FDA-approved Septin9 promoter methylation test, Epi proColon^®^) are non-invasive biomarkers for CRC screening [132]. Integrating mutation, methylation, RNA expression, protein/metabolite, and microbiota data may enable high-precision models for detecting early-stage tumors or minimal residual disease. Machine learning models combining plasma ctDNA mutations, fragmentation patterns, methylation features, and circulating proteins aim to further improve early detection rates.

## 9. Novel Therapeutic Strategies

Metabolic and epigenetic abnormalities in CRC (CRC) are not only pathogenic mechanisms but also crucial therapeutic targets. In recent years, a series of targeted drugs against metabolic pathways and epigenetic enzymes have emerged or entered clinical trials. Concurrently, comprehensive therapeutic strategies jointly targeting metabolism and epigenetics are being unveiled.

### 9.1. Targeting Metabolic Pathways

Capitalizing on the unique metabolic dependencies of CRC cells, developing small-molecule inhibitors to suppress tumor metabolic advantages is a current research focus. Glycolysis inhibitors are a key area. In CRC, inhibitors targeting LDHA, such as oxamate, significantly inhibit glycolysis and reduce ATP production when combined with metformin, thereby suppressing tumor growth. 2-DG, a structural analog of glucose, competitively inhibits hexokinase, blocks glycolysis, reduces acetyl-CoA generation, and increases histone deacetylase activity [133]. 2-DG has undergone Phase I/II trials in various solid tumors. In CRC, combined with radiotherapy, it significantly modulates DNA damage repair and protein phosphorylation, enhancing radiotherapy efficacy. Another inhibitor, 3-bromopyruvate (3-BrPA), also inhibits hexokinase activity, reduces ATP production, and suppresses cell proliferation in CRC models [134]. Drugs targeting glutamine metabolism, such as Compound 968 (a GLS1 inhibitor), have shown antitumor effects in preclinical models [135]. Additionally, fatty acid synthesis inhibitors like the FASN inhibitor TVB-2640 and glutathione synthesis inhibitors like BSO can reduce tumor reliance on lipids and antioxidants, improving immunotherapy efficacy [136]. Cerulenin specifically inhibits fatty acid synthase (FASN), blocking tumor lipid synthesis, downregulating Wnt/β-catenin and IGF-1 signaling, inducing apoptosis, and inhibiting metastasis. Notably, its combination with oxaliplatin synergistically enhances antitumor activity, reduces chemotherapy toxicity, and significantly suppresses CRC liver metastasis [137]. Bezafibrate activates fatty acid oxidation (FAO), inducing tumor cells to secrete CXCL9/CXCL10 chemokines and promoting CD8^+^ T cell infiltration. When combined with anti-PD-1, it significantly boosts immunotherapy efficacy by upregulating CXCR3 expression [138]. Currently, the clinical application of metabolic-targeted drugs in solid tumors remains in early stages, with limited monotherapy efficacy. However, they offer potential for exploiting metabolic vulnerabilities. Future success will hinge on selecting specific patient subgroups and optimizing combination therapies. Here, we summarize relevant clinical trials targeting metabolic pathways (Table 3).

### 9.2. Targeting Epigenetic Therapies

Epigenetic drugs reshape gene expression to inhibit tumorigenesis and progression. Although monotherapy efficacy in CRC is less pronounced than in leukemia, several agents are under clinical evaluation. DNMT inhibitors like 5-azacytidine (5-azaC) and decitabine incorporate into DNA and covalently bind DNMTs, leading to passive dilution of DNA methylation and reactivation of silenced tumor suppressor genes [139]. Both drugs have been tested in advanced solid tumors, 5-azaC showed safety and some disease control in a Phase II trial for metastatic CRC [140]. Zebularine, an orally available DNMTi with higher selectivity and lower toxicity, is also promising for CRC. Decitabine reactivates methylated tumor suppressors by inhibiting DNMTs [141]. Combined with 5-FU or oxaliplatin, it synergistically sensitizes chemotherapy. However, in CIMP-high subtypes, low doses may promote proliferation via BCL2 restoration, necessitating strict dose control. HDAC inhibitors targeting classes I/II/IV, such as vorinostat, belinostat, and trichostatin A, relieve epigenetic repression and increase tumor suppressor gene expression [142]. In CRC, belinostat has entered Phase I trials [143]. Vorinostat, a pan-HDAC inhibitor, primarily reverses oxaliplatin and 5-FU resistance by downregulating thymidylate synthase expression. Romidepsin selectively inhibits HDAC1/2, modulates the Treg/Th1 balance in the tumor microenvironment, and significantly suppresses CRC progression when combined with immune checkpoint inhibitors (ICIs) [144]. Histone methyltransferase inhibitors are mostly preclinical, EZH2 inhibitors may be effective in CRC subtypes with EZH2 overexpression/mutations [145]. Sirtuins, as NAD^+^-dependent deacetylase targets, are also of interest [45]. Tenovin-6 inhibits SIRT1/2, inducing tumor cell cycle arrest. Natural long-chain fatty acids activate SIRT6, inhibiting glycolysis and CRC progression [146]. Noncoding RNA therapies are emerging, using miRNA mimics to restore lost tumor-suppressive miRNA function or antisense/sponge molecules to inhibit oncogenic miRNAs. In CRC, upregulating miR-143/145, miR-34 or downregulating miR-135b, miR-21 suppresses proliferation and invasion in cell and animal models [147]. A miR-16 mimic has undergone Phase I trials in non-small cell lung cancer, validating miRNA therapy feasibility [148]. Antisense oligonucleotides against miR-135b significantly inhibit tumors in CRC preclinical models [149]. Therapies targeting lncRNAs and circRNAs remain exploratory due to broad targeting, but advances in delivery systems (e.g., nanoparticles, viral vectors) and specificity enhancement may position them as future epigenetic treatment avenues.

Across solid tumors, many metabolic agents demonstrate pharmacodynamic target engagement yet limited monotherapy benefit in time-to-event endpoints. For example, the mitochondrial metabolism modulator devimistat failed to improve overall survival versus standard FOLFIRINOX in a phase III study, and the glutaminase inhibitor telaglenastat did not improve outcomes when added to cabozantinib in metastatic renal-cell carcinoma despite tolerability in phase I, highlighting challenges in converting metabolic target engagement into survival gains [150].

Class-specific toxicities often narrow the therapeutic window. Complex I inhibition with IACS-010759 was limited by elevated blood lactate, lactic acidosis, vomiting, and peripheral neuropathy that constrained dose intensity, whereas histone deacetylase inhibitors have repeatedly shown fatigue, gastrointestinal toxicity, cytopenias, and QT concerns in early-phase studies [151]. Fatty-acid synthase inhibition with denifanstat has been generally tolerable but can be associated with metabolic and dermatologic effects across indications, underscoring the need for proactive safety monitoring.

Translational barriers include metabolic plasticity and pathway redundancies that enable rapid compensatory rewiring, imperfect patient selection due to immature predictive biomarkers, and difficulty sustaining target-inhibitory exposures without off-target metabolic stress. Arginine-pathway modulation illustrates these issues: the arginase inhibitor INCB001158 showed acceptable safety but limited antitumor activity as monotherapy or with PD-1 blockade, and historical DHODH inhibition with brequinar failed to translate in solid tumors, despite clear on-target effects [152]. These experiences argue for biomarker-anchored enrichment, pharmacodynamic verification of pathway suppression in tumor tissue or ctDNA, and mechanism-based combinations that avoid overlapping toxicities.

Trials should incorporate prospective biomarkers of dependency or vulnerability, prespecify pharmacodynamic readouts to confirm in-tumor pathway modulation, and prioritize combinations with chemotherapy, anti-angiogenic therapy, or immunotherapy only when mechanistic complementarity and safety are demonstrated in phase I. Adaptive and enrichment designs may mitigate heterogeneity and improve the probability of detecting clinically meaningful benefit.

### 9.3. Therapeutic Targeted Delivery of ncRNAs That Modulate Metabolic Pathways in CRC

Metabolic reprogramming is a core phenotype of colorectal cancer, encompassing enhanced glycolysis, lactate efflux, activated de novo lipogenesis, and upregulated one-carbon metabolism. Building interventional noncoding RNA therapeutics around these pathways and achieving tumor-selective accumulation, intracellular release, and endosomal escape through tissue- and cell-specific delivery systems are pivotal for translation. The principal carrier platforms include clinically scalable lipid nanoparticles, polymeric nanoparticles with programmable surfaces, and engineered exosomes with homing properties [153]. Ligand decoration can be layered on to improve selectivity and tissue penetration. Across multiple preclinical CRC studies, these platforms have shown the capacity to “de-reprogram” metabolic circuits, sensitize chemotherapy, and suppress metastasis.

At the level of glycolysis and lactate metabolism, small-interfering RNA targeting lactate dehydrogenase A, delivered via cationic polymer complexes, reduces tumor-cell lactate secretion, prevents M2-like polarization of tumor-associated macrophages, and converts protective autophagy into lethal autophagy, thereby markedly enhancing the antitumor efficacy of oxaliplatin [154]. This underscores the feasibility of combining metabolic pathway intervention with chemotherapy. Another study employed biodegradable poly(lactic-co-glycolic acid) nanoparticles to deliver siRNA against Notch1, reducing glycolytic flux through the PCAF and HIF-1α axis, mitigating 5-fluorouracil resistance, and inducing pyroptosis, with efficacy demonstrated in both cellular and murine models. This highlights the value of RNA intervention at signaling–metabolism coupling nodes.

Focusing on metabolic microenvironment remodeling and metastasis suppression, hepatocyte-directed delivery of miR-122 produced prophylactic and survival benefits in models of colorectal cancer liver metastasis. Galactose-decorated lipid–calcium phosphate nanoparticles enabled efficient and selective delivery of miR-122 to hepatocytes, significantly reducing metastatic burden and prolonging survival across multiple models, thereby supporting an organ-specific metabolic correction strategy [155]. Notably, tumor-derived exosomal circular RNAs can upregulate glycolysis via the miR-122–PKM2 axis and drive oxaliplatin resistance; this “natural delivery” mechanism conversely suggests the therapeutic potential of using exosomes to deliver antagonistic miRNAs or siRNAs to reverse that axis.

Engineered exosomes and ligand modification enable tissue- and cell-specific targeting, improving the therapeutic index of ncRNAs in colorectal cancer. Targeted exosomes produced from donor cells expressing a TM4SF5-binding peptide efficiently loaded and delivered miRNAs that suppress MACC1, achieving stronger tumor inhibition and superior in vivo accumulation in xenograft models, thus demonstrating the feasibility of biomarker-guided targeted delivery [156]. In addition, aptamers, as small nucleic acid ligands, selectively recognize tumor surface receptors and can act as “molecular towlines” for miRNA or siRNA, improving internalization and pharmacologic effect in multiple tumor models; this offers a readily transferable design for metabolic pathway–targeted delivery in CRC.

Advancing from platform to clinic requires coordinated progress along three axes. First is patient stratification and pharmacodynamic readouts: metabolic fingerprints such as a high-lactate phenotype, elevated glycolytic scores, or increased LDHA activity can guide enrollment and response assessment. Concomitant tissue or liquid-biopsy measurements of target knockdown, glycolytic flux, and lactate burden should verify on-target mechanism in vivo. Second is delivery safety and distribution control: lipid and polymeric nanoparticles already have manufacturing maturity, but terminal surface modifications and particle size must be optimized in CRC to enhance tumor penetration and intratumoral distribution uniformity [157]. Engineered exosomes offer homing and immunocompatibility advantages, yet standardization and batch consistency remain bottlenecks for industrialization. Third is rational combination design: pairing RNA-based metabolic interventions with chemotherapy, anti-angiogenic therapy, or immunotherapy should be grounded in mechanistic complementarity and non-overlapping toxicities, with prospective biomarker-controlled studies prioritized to delineate clinical net benefit and safety margins [158].

Targeted delivery of ncRNAs that modulate metabolic pathways in colorectal cancer is supported by strong biological rationale and encouraging preclinical feasibility. Lipid and polymer platforms provide high payload capacity and scalable manufacturability, whereas engineered exosomes and aptamers afford higher tissue and cell specificity. Representative studies targeting LDHA, PKM2, HIF-1α, and Notch1 have demonstrated multiple gains, including chemosensitization, antimetastatic activity, and reversal of drug resistance. The next phase should emphasize metabolic fingerprint–guided patient selection, quantifiable pharmacodynamic validation, and robust multi-center external replication, to advance this strategy from mechanistic evidence to clinical trials and standardized translation.

## 10. Limitations and Challenges for Clinical Use of Multi-Omics and Liquid Biopsy

Although multi-omics profiling and liquid biopsy are reshaping cancer diagnostics, several practical barriers limit immediate, large-scale clinical adoption. From a cost standpoint, comprehensive multi-omics assays that combine genomics, epigenomics, proteomics, metabolomics and microbiomics require specialized instrumentation, dedicated bioinformatics pipelines and repeated sampling, which increases per-patient costs and complicates reimbursement. For liquid biopsy, downstream diagnostic cascades triggered by false positives in screening settings can further inflate system-level expenditures; heterogeneous payer coverage compounds these disparities across regions and institutions. These economic considerations argue for biomarker-guided use, comparative-effectiveness analyses and clearer reimbursement frameworks before routine deployment.

Reproducibility remains a central challenge. Analytical performance varies with pre-analytical variables such as tube type, time-to-plasma, cellular lysis and plasma volume, and with assay-specific limits of detection at low variant allele fractions, particularly in early-stage disease or minimal-residual-disease settings. Cross-platform concordance for fusions and copy-number alterations is lower than for single-nucleotide variants, reinforcing the need for orthogonal confirmation with tissue whenever feasible. Community efforts, including FDA-led SEQC2 benchmarking and emerging common lexicons and reference materials for multi-omics integration, have improved standardization. However, assay harmonization, calibrated reporting thresholds and shared quality-control metrics are still prerequisites for robust clinical generalizability.

Accessibility is uneven. Implementing multi-omics and ctDNA workflows requires laboratory accreditation, trained personnel, secure data infrastructure and timely logistics for sample handling, which are not uniformly available across health systems. Recent implementation reviews highlight structural barriers, including limited awareness, variable turnaround times, fragmented care pathways and inequitable access in community settings. Addressing these gaps will require standardized pre-analytical protocols, proficiency testing, transparent reporting of analytical and clinical validity, and prospective studies that demonstrate actionable clinical utility—particularly where guidelines currently recommend caution, such as the routine use of ctDNA to detect molecular residual disease outside clinical trials.

Together, these constraints suggest that the near-term clinical impact of multi-omics and liquid biopsy will depend on focused indications with clear utility, harmonized standards across laboratories, and health-economic models that align reimbursement with measurable patient benefit.

## 11. Conclusions

Colorectal carcinogenesis is a complex multistep process where metabolic reprogramming and epigenetic modifications interact critically to drive malignant progression. Tumor cells reprogram glucose, amino acid, and lipid metabolism to meet their growth needs. At the same time, they utilize epigenetic modifications to regulate gene expression and adapt to environmental stress. These two mechanisms are interconnected: metabolic alterations directly impact epigenetic enzyme activity, reshaping gene expression profiles, while epigenetic changes regulate the expression of key metabolic enzymes and regulators, remodeling cellular metabolism. With breakthroughs in multi-omics technologies, the landscape of metabolism–epigenetics crosstalk is becoming clearer, though key questions remain unresolved. The roles of novel epigenetic modifications in CRC metabolic regulation require further elucidation. Sensitivity mechanisms of different metabolic subtypes to epigenetic drugs need validation through more models and clinical trials. In clinical translation, despite challenges in metabolic and epigenetic therapies for solid tumors, including CRC, novel combination strategies and precision dosing hold promise for overcoming these hurdles. Integrating metabolic reprogramming with epigenetic abnormalities provides a comprehensive perspective for understanding CRC and opens new avenues for diagnosis and therapy. In the near future, precision medicine strategies based on metabolism–epigenetics interactions will likely become integral to CRC management, improving patient prognosis and quality of life.

## Figures and Tables

**Table 1:** The role of different ncRNAs in CRC.

Regulation	ncRNA	Key Mechanism	Impact on CRC	Ref.
↑	miR-21	Suppresses autophagy via PTEN/AKT/TFEB	↑ invasion, ↓ 5-FU sensitivity	[68]
↑	miR-452-5p	Represses PKN2/DUSP6; activates MAPK-ERK	↑ proliferation, chemoresistance	[68]
↑	miR-496	Inhibits RASSF6; activates Wnt signaling	↑ EMT, motility	[69]
↑	miR-298	Targets PTEN; activates AKT/ERK and mTOR	↑ metabolic activity, growth	[69]
↑	miR-645	Targets EFNA5	↑ migration, metastasis	[70]
↓	miR-130a-3p	Targets WNT1; blocks Wnt	↓ growth (cells and xenograft)	[71]
↓	miR-144-5p	Represses RNF187	↓ migration, invasion	[71]
↓	miR-144-3p	Targets BCL6; dampens β-catenin	↓ proliferation, cell cycle	[72]
↓	miR-215-5p	Binds CTNNBIP1→restrains Wnt	↓ clonogenicity, liver metastasis	[72]
↓	miR-148b	p53-induced; targets p55PIK	↓ proliferation, tumor growth	[72]
↓	miR-16	Represses surviving (BIRC5)	↑ apoptosis, ↓ growth	[73]
↓	miR-654-3p	Suppresses SRC	↓ proliferation and invasion; ↑ apoptosis	[73]
↑	lncRNA CTBP1-AS2	Sponges miR-93-5p; TGF-β1/Smad2/3	↑ proliferation and invasion; ↓ apoptosis	[74]
↑	lncRNA COL4A2-AS1	Sponges miR-20b-5p; ↑HIF1A	↑ proliferation and aerobic glycolysis	[74]
↑	lncRNA NEAT1	Sponges miR-34a; ↑ SIRT1 (Wnt)	↑ growth and invasion	[74]
↑	lncRNA RoR	Sponges miR-6833-3p; ↑ SMC4	↑ proliferation; ↓ apoptosis	[74]
↑	lncRNA SNHG8	Sponges miR-588; ↑ ATG7	↑ proliferation and autophagy	[74]
↑	lncRNA CASC21	Sponges miR-7-5p; ↑ YAP1	↑ migration and EMT; ↓ apoptosis	[75]
↑	lncRNA MCF2L-AS1	Sponges miR-874-3p; ↑ CCNE1	↑ proliferation and EMT; ↓ apoptosis	[75]
↑	lncRNA RNCR3	Sponges miR-1301-3p; ↑ AKT1	↑ proliferation and invasion; ↓ apoptosis	[75]
↓	lncRNA MIR503HG	Sponges miR-107; ↑ PAR4	↓ migration and invasion	[76]
↓	lncRNA DPP10-AS1	Sponges miR-127-3p; ↓ ADCY1	↓ stemness and invasion; ↑ apoptosis	[76]
↓	lncRNA MCM3AP-AS1	Sponges miR-19a-3p; ↑ FOXF2	↓ proliferation and migration	[76]

↑: Up; ↓: Down.

**Table 2:** Representative novel diagnostic approaches in CRC: biomarkers, specimens, use cases, and performance metrics.

Study/Approach	Key Biomarkers/Targets	Sample Types	Use Case/Setting	Key Performance Metrics
Multi-omics consensus molecular subtyping (CMS1, CMS3)	Integrated genomic, epigenetic, transcriptomic, proteomic features	Tumor tissue	Molecular subtyping, prognosis, treatment selection	CMS3 (*p* = 0.002)
Plasma cfDNA methylation panel (six-marker example)	BCAN, BCAT1, IKZF1, SEPTIN9_1, SEPTIN9_2, VAV3	Plasma	Noninvasive detection and recurrence risk stratification	Sensitivity: 78.0%; Specificity: 90.2%;
Single-cell RNA sequencing (scRNA-seq) of CRC and adjacent tissues	Cell type–specific metabolic and epigenetic state signatures	Tumor and adjacent tissue	Personalized diagnostic “fingerprints,” prognostic modeling	Model C-index/AUC (if available): 0.86
ctDNA methylation markers and multigene panels	SDC2, TFPI2, NDRG4, SEPTIN9 (and related panels)	Plasma	Noninvasive screening and adjuvant monitoring	Sensitivity: 88%; Specificity: 93.4%
Single-cell sequencing of circulating tumor cells (CTCs)	Whole-genome and metabolic/epigenetic phenotypes at single-cell level	Peripheral blood	Metastatic potential prediction and molecular classification	PFS (*p* < 0.001)
Exosomal nucleic acids and metabolites	Exosomal miRNAs such as miR-21; metabolic enzyme mRNAs/metabolites	Plasma/serum	Diagnostic aid and progression assessment	Sensitivity: 91.1% Specificity: 95.5%
Multi-omics liquid biopsy with AI integration	ctDNA mutations, methylation, fragmentation plus circulating proteins/metabolites	Plasma (optionally combined with stool/clinical data)	Noninvasive early detection and MRD monitoring	Sensitivity: 92%; Specificity: 89.5%
Multi-omics integrative diagnostic framework	Mutations, methylation, RNA expression, proteins/metabolites, microbiome	Tissue and liquid biopsies	Precision diagnosis and early detection	Concordance with pathology/external validation metrics: R = 0.82; AUC = 0.86

**Table 3:** Clinical trials related to targeted metabolic pathways in CRC.

NCT Number	Study Title	Conditions	Interventions	Phases
NCT06944548	Evaluation of the Effect of Adapted Physical Activity on the Modification of Lipid Metabolism During Chemotherapy for Metastatic COLorectal Cancer	Metastatic Colorectal Cancer (CRC)|Volunteer Subjects	BIOLOGICAL: Lipidomic analyses|OTHER: Adapted physical activity program	PHASE2
NCT03831698	Omega 3 Fatty Acids in Colorectal Cancer (CRC) Prevention in Patients With Lynch Syndrome (COLYNE)	Colorectal Cancer|Lynch Syndrome	DRUG: Omega-3 fatty acid ethyl esters (2 g)	PHASE2
NCT06886022	Diagnosis, Treatment, and Prevention of Colorectal Cancer Amid Abnormal Lipid Metabolism	Colorectal Cancer	PROCEDURE: Colorectal cancer procedures	NA
NCT01591590	Correlating the Tumoral Metabolic Progression Index to Patient’s Outcome in Advanced Colorectal Cancer	Colorectal Cancer	OTHER: FDG PET-CT|OTHER: Diffusion MRI|OTHER: Blood samples (plasma preparation and CTC)	NA
NCT02903914	Arginase Inhibitor INCB001158 as a Single Agent and in Combination With Immune Checkpoint Therapy in Patients With Advanced/Metastatic Solid Tumors	Metastatic Cancer|Solid Tumors|Colorectal Cancer (CRC)|Gastric Cancer|Head and Neck Cancer|Lung Cancer|Renal Cell Carcinoma (RCC)|Bladder Cancer|UC (Urothelial Cancer)|Mesothelioma	DRUG: INCB001158|DRUG: Pembrolizumab	PHASE1
NCT03550885	Diet Modulation of Bacterial Sulfur and Bile Acid Metabolism and Colon Cancer Risk	Colorectal Cancer	OTHER: High taurine and saturated fat diet|OTHER: Low in taurine and saturated fat diet	NA
NCT01426490	The Effects of Vitamin B-6 Status on Homocysteine, Oxidative Stress, One-carbon Metabolism and Methylation: Cross-section, Case-control, Intervention and Follow-up Studies in Colorectal Cancer	Colorectal Cancer	DIETARY_SUPPLEMENT: Vitamin C|DIETARY_SUPPLEMENT: Vitamin B6|DIETARY_SUPPLEMENT: Folic acid|DIETARY_SUPPLEMENT: Vitamin B6 plus folic acid	PHASE2|
NCT02699047	Fish Oil Supplementation in Gastrointestinal Cancer	Gastrointestinal Cancer|Colorectal Cancer|Stomach Cancer	DIETARY_SUPPLEMENT: Encapsuled fish oil|DIETARY_SUPPLEMENT: Encapsulated Olive oil	NA
NCT06710314	A Metabolomics-based Study to Explore the Mechanism of Remission of Metabolic Syndrome Radical Resection of Colorectal Cancer	Metabolomics	DIAGNOSTIC_TEST: Colorectal cancer patients with hypertension|DIAGNOSTIC_TEST: Colorectal cancer patients with diabetes|DIAGNOSTIC_TEST: Colorectal cancer patients with fatty liver	NA
NCT05494658	Impact of Preoperative Oral Branched-chain Amino Acids on Reducing Postoperative Insulin Resistance.	Insulin Resistance|Colorectal Cancer	DIETARY_SUPPLEMENT: BCAA|DIETARY_SUPPLEMENT: water	NA

## Data Availability

No applicable.

## References

[1] Murphy C.C., Zaki T.A. (2024). Changing epidemiology of colorectal cancer—Birth cohort effects and emerging risk factors. Nat. Rev. Gastroenterol. Hepatol..

[2] Gupta S., May F.P., Kupfer S.S., Murphy C.C. (2024). Birth Cohort Colorectal Cancer (CRC): Implications for Research and Practice. Clin. Gastroenterol. Hepatol. Off. Clin. Pract. J. Am. Gastroenterol. Assoc..

[3] Wong C.C., Yu J. (2023). Gut microbiota in colorectal cancer development and therapy. Nat. Rev. Clin. Oncol..

[4] Mao Y., Xia Z., Xia W., Jiang P. (2024). Metabolic reprogramming, sensing, and cancer therapy. Cell Rep..

[5] Liu Y., Zhao Y., Song H., Li Y., Liu Z., Ye Z., Zhao J., Wu Y., Tang J., Yao M. (2024). Metabolic reprogramming in tumor immune microenvironment: Impact on immune cell function and therapeutic implications. Cancer Lett..

[6] Wang J., He Y., Hu F., Hu C., Sun Y., Yang K., Yang S. (2024). Metabolic Reprogramming of Immune Cells in the Tumor Microenvironment. Int. J. Mol. Sci..

[7] Avella Patino D.M., Radhakrishnan V., Suvilesh K.N., Manjunath Y., Li G., Kimchi E.T., Staveley-O’Carroll K.F., Warren W.C., Kaifi J.T., Mitchem J.B. (2022). Epigenetic Regulation of Cancer Immune Cells. Semin. Cancer Biol..

[8] Li C., Chen K., Fang Q., Shi S., Nan J., He J., Yin Y., Li X., Li J., Hou L. (2024). Crosstalk between epitranscriptomic and epigenomic modifications and its implication in human diseases. Cell Genom..

[9] Roy S., Deka D., Kondaveeti S.B., Ayyadurai P., Siripragada S., Philip N., Pathak S., Duttaroy A.K., Banerjee A. (2025). An overview of potential of natural compounds to regulate epigenetic modifications in colorectal cancer: A recent update. Epigenetics.

[10] Guo L., Lee Y.T., Zhou Y., Huang Y. (2022). Targeting epigenetic regulatory machinery to overcome cancer therapy resistance. Semin. Cancer Biol..

[11] Chen Z., Natarajan R. (2022). Epigenetic modifications in metabolic memory: What are the memories, and can we erase them?. Am. J. Physiol. Cell Physiol..

[12] Yue S.W., Liu H.L., Su H.F., Luo C., Liang H.F., Zhang B.X., Zhang W. (2023). m6A-regulated tumor glycolysis: New advances in epigenetics and metabolism. Mol. Cancer.

[13] Babar Q., Saeed A., Tabish T.A., Pricl S., Townley H., Thorat N. (2022). Novel epigenetic therapeutic strategies and targets in cancer. Biochim. Biophys. Acta Mol. Basis Dis..

[14] Sun L., Zhang H., Gao P. (2022). Metabolic reprogramming and epigenetic modifications on the path to cancer. Protein Cell.

[15] Huang C.Y., Huang C.Y., Pai Y.C., Lin B.R., Lee T.C., Liang P.H., Yu L.C. (2019). Glucose Metabolites Exert Opposing Roles in Tumor Chemoresistance. Front. Oncol..

[16] Liu H., Liang Z., Zhou C., Zeng Z., Wang F., Hu T., He X., Wu X., Wu X., Lan P. (2021). Mutant KRAS triggers functional reprogramming of tumor-associated macrophages in colorectal cancer. Signal Transduct. Target. Ther..

[17] Huang Y., Xiong C., Wang C., Deng J., Zuo Z., Wu H., Xiong J., Wu X., Lu H., Hao Q. (2023). p53-responsive CMBL reprograms glucose metabolism and suppresses cancer development by destabilizing phosphofructokinase PFKP. Cell Rep..

[18] Jing Z., Liu Q., He X., Jia Z., Xu Z., Yang B., Liu P. (2022). NCAPD3 enhances Warburg effect through c-myc and E2F1 and promotes the occurrence and progression of colorectal cancer. J. Exp. Clin. Cancer Res..

[19] Zhu G., Pei L., Xia H., Tang Q., Bi F. (2021). Role of oncogenic KRAS in the prognosis, diagnosis and treatment of colorectal cancer. Mol. Cancer.

[20] Zhou M., He J., Li Y., Jiang L., Ran J., Wang C., Ju C., Du D., Xu X., Wang X. (2023). N^6^-methyladenosine modification of REG1α facilitates colorectal cancer progression via β-catenin/MYC/LDHA axis mediated glycolytic reprogramming. Cell Death Dis..

[21] Padder R.A., Bhat Z.I., Ahmad Z., Singh N., Husain M. (2020). DRP1 Promotes BRAF^V600E^-Driven Tumor Progression and Metabolic Reprogramming in Colorectal Cancer. Front. Oncol..

[22] Guan Y., Yao W., Yu H., Feng Y., Zhao Y., Zhan X., Wang Y. (2023). Chronic stress promotes colorectal cancer progression by enhancing glycolysis through β2-AR/CREB1 signal pathway. Int. J. Biol. Sci..

[23] Barisciano G., Colangelo T., Rosato V., Muccillo L., Taddei M.L., Ippolito L., Chiarugi P., Galgani M., Bruzzaniti S., Matarese G. (2020). miR-27a is a master regulator of metabolic reprogramming and chemoresistance in colorectal cancer. Br. J. Cancer.

[24] Sun H., Zhang C., Zheng Y., Liu C., Wang X., Cong X. (2022). Glutamine deficiency promotes recurrence and metastasis in colorectal cancer through enhancing epithelial-mesenchymal transition. J. Transl. Med..

[25] Yu W., Huang J., Dong Q., Li W., Jiang L., Zhang Q., Sun L., Yuan S., He X. (2022). Ag120-Mediated Inhibition of ASCT2-Dependent Glutamine Transport has an Anti-Tumor Effect on Colorectal Cancer Cells. Front. Pharmacol..

[26] Han Y., Pu Y., Liu X., Liu Z., Chen Y., Tang L., Zhou J., Song Q., Ji Q. (2024). YTHDF1 regulates GID8-mediated glutamine metabolism to promote colorectal cancer progression in m6A-dependent manner. Cancer Lett..

[27] Hua Q., Zhang B., Xu G., Wang L., Wang H., Lin Z., Yu D., Ren J., Zhang D., Zhao L. (2021). CEMIP, a novel adaptor protein of OGT, promotes colorectal cancer metastasis through glutamine metabolic reprogramming via reciprocal regulation of β-catenin. Oncogene.

[28] Lin Z., Yang S., Qiu Q., Cui G., Zhang Y., Yao M., Li X., Chen C., Gu J., Wang T. (2024). Hypoxia-induced cysteine metabolism reprogramming is crucial for the tumorigenesis of colorectal cancer. Redox Biol..

[29] Chen Z., Xu J., Fang K., Jiang H., Leng Z., Wu H., Zhang Z., Wang Z., Li Z., Sun M. (2025). FOXC1-mediated serine metabolism reprogramming enhances colorectal cancer growth and 5-FU resistance under serine restriction. Cell Commun. Signal. CCS.

[30] Li J., Song P., Jiang T., Dai D., Wang H., Sun J., Zhu L., Xu W., Feng L., Shin V.Y. (2018). Heat Shock Factor 1 Epigenetically Stimulates Glutaminase-1-Dependent mTOR Activation to Promote Colorectal Carcinogenesis. Mol. Ther. J. Am. Soc. Gene Ther..

[31] Lu L., Zhang Q., Aladelokun O., Berardi D., Shen X., Marin A., Garcia-Milian R., Roper J., Khan S.A., Johnson C.H. (2025). Asparagine synthetase and G-protein coupled estrogen receptor are critical responders to nutrient supply in KRAS mutant colorectal cancer. Int. J. Cancer.

[32] Pan Q., Yu F., Jin H., Zhang P., Huang X., Peng J., Xie X., Li X., Ma N., Wei Y. (2023). eIF3f Mediates SGOC Pathway Reprogramming by Enhancing Deubiquitinating Activity in Colorectal Cancer. Adv. Sci..

[33] Toda K., Kawada K., Iwamoto M., Inamoto S., Sasazuki T., Shirasawa S., Hasegawa S., Sakai Y. (2016). Metabolic Alterations Caused by KRAS Mutations in Colorectal Cancer Contribute to Cell Adaptation to Glutamine Depletion by Upregulation of Asparagine Synthetase. Neoplasia.

[34] Nenkov M., Ma Y., Gaßler N., Chen Y. (2021). Metabolic Reprogramming of Colorectal Cancer Cells and the Microenvironment: Implication for Therapy. Int. J. Mol. Sci..

[35] Chen D., Zhou X., Yan P., Yang C., Li Y., Han L., Ren X. (2023). Lipid metabolism reprogramming in colorectal cancer. J. Cell. Biochem..

[36] Dong C., Zhang Y., Zeng J., Chong S., Liu Y., Bian Z., Fan S., Chen X. (2024). FUT2 promotes colorectal cancer metastasis by reprogramming fatty acid metabolism via YAP/TAZ signaling and SREBP-1. Commun. Biol..

[37] Zhao Y., Liu M.J., Zhang L., Yang Q., Sun Q.H., Guo J.R., Lei X.Y., He K.Y., Li J.Q., Yang J.Y. (2024). High mobility group A1 (HMGA1) promotes the tumorigenesis of colorectal cancer by increasing lipid synthesis. Nat. Commun..

[38] Liu X., Lu J., Ni X., He Y., Wang J., Deng Z., Zhang G., Shi T., Chen W. (2025). FASN promotes lipid metabolism and progression in colorectal cancer via the SP1/PLA2G4B axis. Cell Death Discov..

[39] Yang Y., He J., Zhang B., Zhang Z., Jia G., Liu S., Wu T., He X., Wang N. (2021). SLC25A1 promotes tumor growth and survival by reprogramming energy metabolism in colorectal cancer. Cell Death Dis..

[40] Zhang Z., Gao Y., Qian Y., Wei B., Jiang K., Sun Z., Zhang F., Yang M., Baldi S., Yu X. (2025). The Lyn/RUVBL1 Complex Promotes Colorectal Cancer Liver Metastasis by Regulating Arachidonic Acid Metabolism Through Chromatin Remodeling. Adv. Sci..

[41] Leiphrakpam P.D., Are C. (2024). PI3K/Akt/mTOR Signaling Pathway as a Target for Colorectal Cancer Treatment. Int. J. Mol. Sci..

[42] Wang H., Chen Y., Liu Y., Li Q., Luo J., Wang L., Chen Y., Sang C., Zhang W., Ge X. (2022). The lncRNA ZFAS1 regulates lipogenesis in colorectal cancer by binding polyadenylate-binding protein 2 to stabilize SREBP1 mRNA. Mol. Ther. Nucleic Acids.

[43] Pranzini E., Pardella E., Muccillo L., Leo A., Nesi I., Santi A., Parri M., Zhang T., Uribe A.H., Lottini T. (2022). SHMT2-mediated mitochondrial serine metabolism drives 5-FU resistance by fueling nucleotide biosynthesis. Cell Rep..

[44] Schwartz A.J., Goyert J.W., Solanki S., Kerk S.A., Chen B., Castillo C., Hsu P.P., Do B.T., Singhal R., Dame M.K. (2021). Hepcidin sequesters iron to sustain nucleotide metabolism and mitochondrial function in colorectal cancer epithelial cells. Nat. Metab..

[45] Wang H.L., Chen Y., Wang Y.Q., Tao E.W., Tan J., Liu Q.Q., Li C.M., Tong X.M., Gao Q.Y., Hong J. (2022). Sirtuin5 protects colorectal cancer from DNA damage by keeping nucleotide availability. Nat. Commun..

[46] Liu J., Li H., Wang L., Wang S., Tang Q. (2024). Spatial transcriptome and single-cell reveal the role of nucleotide metabolism in colorectal cancer progression and tumor microenvironment. J. Transl. Med..

[47] Demetriadou C., Raoukka A., Charidemou E., Mylonas C., Michael C., Parekh S., Koufaris C., Skourides P., Papageorgis P., Tessarz P. (2022). Histone N-terminal acetyltransferase NAA40 links one-carbon metabolism to chemoresistance. Oncogene.

[48] Li G., Liu H., Yu Y., Wang Q., Yang C., Yan Y., Wang F., Mao Y. (2024). Desulfovibrio desulfuricans and its derived metabolites confer resistance to FOLFOX through METTL3. EBioMedicine.

[49] Kim J., Cho Y.A., Kim D.H., Lee B.H., Hwang D.Y., Jeong J., Lee H.J., Matsuo K., Tajima K., Ahn Y.O. (2012). Dietary intake of folate and alcohol, MTHFR C677T polymorphism, and colorectal cancer risk in Korea. Am. J. Clin. Nutr..

[50] Wei W., Qin B., Wen W., Zhang B., Luo H., Wang Y., Xu H., Xie X., Liu S., Jiang X. (2023). FBXW7β loss-of-function enhances FASN-mediated lipogenesis and promotes colorectal cancer growth. Signal Transduct. Target. Ther..

[51] Mattei A.L., Bailly N., Meissner A. (2022). DNA methylation: A historical perspective. Trends Genet..

[52] Lee A.V., Nestler K.A., Chiappinelli K.B. (2024). Therapeutic targeting of DNA methylation alterations in cancer. Pharmacol. Ther..

[53] Hu Y.H., Ma S., Zhang X.N., Zhang Z.Y., Zhu H.F., Ji Y.H., Li J., Qian X.L., Wang Y.X. (2019). Hypermethylation Of ADHFE1 Promotes The Proliferation Of Colorectal Cancer Cell Via Modulating Cell Cycle Progression. OncoTargets Ther..

[54] Suzuki H., Watkins D.N., Jair K.W., Schuebel K.E., Markowitz S.D., Chen W.D., Pretlow T.P., Yang B., Akiyama Y., Van Engeland M. (2004). Epigenetic inactivation of SFRP genes allows constitutive WNT signaling in colorectal cancer. Nat. Genet..

[55] Park P.H., Keith K., Calendo G., Jelinek J., Madzo J., Gharaibeh R.Z., Ghosh J., Sapienza C., Jobin C., Issa J.J. (2024). Association between gut microbiota and CpG island methylator phenotype in colorectal cancer. Gut Microbes.

[56] Salem M.E., Bodor J.N., Puccini A., Xiu J., Goldberg R.M., Grothey A., Korn W.M., Shields A.F., Worrilow W.M., Kim E.S. (2020). Relationship between MLH1, PMS2, MSH2 and MSH6 gene-specific alterations and tumor mutational burden in 1057 microsatellite instability-high solid tumors. Int. J. Cancer.

[57] Kawaguchi K., Ohashi T., Kobayashi N., Kanemoto K., Nose M., Shinozaki R., Kataoka T., Fujii H. (2023). Aberrant DNA methylation-mediated NF-κB/fatty acid-binding protein 5 (FABP5) feed-forward loop promotes malignancy of colorectal cancer cells. Biochim. Biophys. Acta Mol. Cell Biol. Lipids.

[58] Tan X., Chen H. (2023). Association between MTHFR gene C677T polymorphism and gestational diabetes mellitus in Chinese population: A meta-analysis. Front. Endocrinol..

[59] Kim Y.I., Pogribny I.P., Basnakian A.G., Miller J.W., Selhub J., James S.J., Mason J.B. (1997). Folate deficiency in rats induces DNA strand breaks and hypomethylation within the p53 tumor suppressor gene. Am. J. Clin. Nutr..

[60] Kim Y.I., Christman J.K., Fleet J.C., Cravo M.L., Salomon R.N., Smith D., Ordovas J., Selhub J., Mason J.B. (1995). Moderate folate deficiency does not cause global hypomethylation of hepatic and colonic DNA or c-myc-specific hypomethylation of colonic DNA in rats. Am. J. Clin. Nutr..

[61] He W., Li Q., Li X. (2023). Acetyl-CoA regulates lipid metabolism and histone acetylation modification in cancer. Biochim. Biophys. Acta Rev. Cancer.

[62] Yao W., Hu X., Wang X. (2024). Crossing epigenetic frontiers: The intersection of novel histone modifications and diseases. Signal Transduct. Target. Ther..

[63] Guan X., Liu R., Wang B., Xiong R., Cui L., Liao Y., Ruan Y., Fang L., Lu X., Yu X. (2024). Inhibition of HDAC2 sensitises antitumour therapy by promoting NLRP3/GSDMD-mediated pyroptosis in colorectal cancer. Clin. Transl. Med..

[64] Yao B., Gui T., Zeng X., Deng Y., Wang Z., Wang Y., Yang D., Li Q., Xu P., Hu R. (2021). PRMT1-mediated H4R3me2a recruits SMARCA4 to promote colorectal cancer progression by enhancing EGFR signaling. Genome Med..

[65] Liu X., Zhang H., Fan Y., Cai D., Lei R., Wang Q., Li Y., Shen L., Gu Y., Zhang Q. (2024). SNORA28 Promotes Proliferation and Radioresistance in Colorectal Cancer Cells through the STAT3 Pathway by Increasing H3K9 Acetylation in the LIFR Promoter. Adv. Sci..

[66] Yang Z., Su W., Zhang Q., Niu L., Feng B., Zhang Y., Huang F., He J., Zhou Q., Zhou X. (2025). Lactylation of HDAC1 Confers Resistance to Ferroptosis in Colorectal Cancer. Adv. Sci..

[67] Nemeth K., Bayraktar R., Ferracin M., Calin G.A. (2024). Non-coding RNAs in disease: From mechanisms to therapeutics. Nat. Rev. Genet..

[68] Chen B., Dragomir M.P., Yang C., Li Q., Horst D., Calin G.A. (2022). Targeting non-coding RNAs to overcome cancer therapy resistance. Signal Transduct. Target. Ther..

[69] Liu C., Rokavec M., Huang Z., Hermeking H. (2023). Curcumin activates a ROS/KEAP1/NRF2/miR-34a/b/c cascade to suppress colorectal cancer metastasis. Cell Death Differ..

[70] Wang D., Liu Q., Ren Y., Zhang Y., Wang X., Liu B. (2021). Association analysis of miRNA-related genetic polymorphisms in miR-143/145 and KRAS with colorectal cancer susceptibility and survival. Biosci. Rep..

[71] Chen W.S., Leung C.M., Pan H.W., Hu L.Y., Li S.C., Ho M.R., Tsai K.W. (2012). Silencing of miR-1-1 and miR-133a-2 cluster expression by DNA hypermethylation in colorectal cancer. Oncol. Rep..

[72] Li J., Zhao L.M., Zhang C., Li M., Gao B., Hu X.H., Cao J., Wang G.Y. (2020). The lncRNA FEZF1-AS1 Promotes the Progression of Colorectal Cancer Through Regulating OTX1 and Targeting miR-30a-5p. Oncol. Res..

[73] Li P., Fan J.B., Gao Y., Zhang M., Zhang L., Yang N., Zhao X. (2016). miR-135b-5p inhibits LPS-induced TNFα production via silencing AMPK phosphatase Ppm1e. Oncotarget.

[74] Ozawa T., Matsuyama T., Toiyama Y., Takahashi N., Ishikawa T., Uetake H., Yamada Y., Kusunoki M., Calin G., Goel A. (2017). CCAT1 and CCAT2 long noncoding RNAs, located within the 8q.24.21 ‘gene desert’, serve as important prognostic biomarkers in colorectal cancer. Ann. Oncol. Off. J. Eur. Soc. Med. Oncol..

[75] Tang J., Yan T., Bao Y., Shen C., Yu C., Zhu X., Tian X., Guo F., Liang Q., Liu Q. (2019). LncRNA GLCC1 promotes colorectal carcinogenesis and glucose metabolism by stabilizing c-Myc. Nat. Commun..

[76] Wang Y., Lu J.H., Wu Q.N., Jin Y., Wang D.S., Chen Y.X., Liu J., Luo X.J., Meng Q., Pu H.Y. (2019). LncRNA LINRIS stabilizes IGF2BP2 and promotes the aerobic glycolysis in colorectal cancer. Mol. Cancer.

[77] Xiong L., Liu H.S., Zhou C., Yang X., Huang L., Jie H.Q., Zeng Z.W., Zheng X.B., Li W.X., Liu Z.Z. (2023). A novel protein encoded by circINSIG1 reprograms cholesterol metabolism by promoting the ubiquitin-dependent degradation of INSIG1 in colorectal cancer. Mol. Cancer.

[78] Liang Z.X., Liu H.S., Xiong L., Yang X., Wang F.W., Zeng Z.W., He X.W., Wu X.R., Lan P. (2021). A novel NF-κB regulator encoded by circPLCE1 inhibits colorectal carcinoma progression by promoting RPS3 ubiquitin-dependent degradation. Mol. Cancer.

[79] Sun S., Li C., Cui K., Liu B., Zhou M., Cao Y., Zhang J., Bian Z., Fei B., Huang Z. (2021). Hsa_circ_0062682 Promotes Serine Metabolism and Tumor Growth in Colorectal Cancer by Regulating the miR-940/PHGDH Axis. Front. Cell Dev. Biol..

[80] He J., Chu Z., Lai W., Lan Q., Zeng Y., Lu D., Jin S., Xu H., Su P., Yin D. (2021). Circular RNA circHERC4 as a novel oncogenic driver to promote tumor metastasis via the miR-556-5p/CTBP2/E-cadherin axis in colorectal cancer. J. Hematol. Oncol..

[81] Li Q., Wang Y., Wu S., Zhou Z., Ding X., Shi R., Thorne R.F., Zhang X.D., Hu W., Wu M. (2019). CircACC1 Regulates Assembly and Activation of AMPK Complex under Metabolic Stress. Cell Metab..

[82] Liu X., Liu Y., Liu Z., Lin C., Meng F., Xu L., Zhang X., Zhang C., Zhang P., Gong S. (2021). CircMYH9 drives colorectal cancer growth by regulating serine metabolism and redox homeostasis in a p53-dependent manner. Mol. Cancer.

[83] Wang Z., Long H., Chang C., Zhao M., Lu Q. (2018). Crosstalk between metabolism and epigenetic modifications in autoimmune diseases: A comprehensive overview. Cell. Mol. Life Sci..

[84] Pascale R.M., Simile M.M., Calvisi D.F., Feo C.F., Feo F. (2022). S-Adenosylmethionine: From the Discovery of Its Inhibition of Tumorigenesis to Its Use as a Therapeutic Agent. Cells.

[85] Zsigrai S., Kalmár A., Nagy Z.B., Barták B.K., Valcz G., Szigeti K.A., Galamb O., Dankó T., Sebestyén A., Barna G. (2020). S-Adenosylmethionine Treatment of Colorectal Cancer Cell Lines Alters DNA Methylation, DNA Repair and Tumor Progression-Related Gene Expression. Cells.

[86] Zhang Y., Yu H., Zhang J., Gao H., Wang S., Li S., Wei P., Liang J., Yu G., Wang X. (2021). Cul4A-DDB1-mediated monoubiquitination of phosphoglycerate dehydrogenase promotes colorectal cancer metastasis via increased S-adenosylmethionine. J. Clin. Investig..

[87] Guertin D.A., Wellen K.E. (2023). Acetyl-CoA metabolism in cancer. Nat. Rev. Cancer.

[88] Anderson G. (2020). Tumour Microenvironment: Roles of the Aryl Hydrocarbon Receptor, O-GlcNAcylation, Acetyl-CoA and Melatonergic Pathway in Regulating Dynamic Metabolic Interactions across Cell Types-Tumour Microenvironment and Metabolism. Int. J. Mol. Sci..

[89] Budagyan K., Cannon A.C., Chatoff A., Benton D., Kurimchak A.M., Araiza-Olivera D., Gerasimova A., Snyder N.W., Duncan J.S., Uribe-Alvarez C. (2025). KRAS G12V mutation-selective requirement for ACSS2 in colorectal adenoma formation. Cell Rep..

[90] Eniafe J., Jiang S. (2021). The functional roles of TCA cycle metabolites in cancer. Oncogene.

[91] Pianka S.T., Li T., Prins T.J., Eldred B.S.C., Kevan B.M., Liang H., Zapanta Rinonos S., Kornblum H.I., Nathanson D.A., Pellegrini M. (2024). D-2-HG Inhibits IDH1mut Glioma Growth via FTO Inhibition and Resultant m6A Hypermethylation. Cancer Res. Commun..

[92] Hu S.S., Han Y., Tan T.Y., Chen H., Gao J.W., Wang L., Yang M.H., Zhao L., Wang Y.Q., Ding Y.Q. (2023). SLC25A21 downregulation promotes KRAS-mutant colorectal cancer progression by increasing glutamine anaplerosis. JCI Insight.

[93] Navas L.E., Carnero A. (2022). Nicotinamide Adenine Dinucleotide (NAD) Metabolism as a Relevant Target in Cancer. Cells.

[94] Dong W., Lu J., Li Y., Zeng J., Du X., Yu A., Zhao X., Chi F., Xi Z., Cao S. (2024). SIRT1: A novel regulator in colorectal cancer. Biomed. Pharmacother..

[95] Pozzi V., Campagna R., Sartini D., Emanuelli M. (2022). Nicotinamide N-Methyltransferase as Promising Tool for Management of Gastrointestinal Neoplasms. Biomolecules.

[96] Campagna R., Mazzanti L., Pompei V., Alia S., Vignini A., Emanuelli M. (2024). The Multifaceted Role of Endothelial Sirt1 in Vascular Aging: An Update. Cells.

[97] Ulanovskaya O.A., Zuhl A.M., Cravatt B.F. (2013). NNMT promotes epigenetic remodeling in cancer by creating a metabolic methylation sink. Nat. Chem. Biol..

[98] van Haren M.J., Gao Y., Buijs N., Campagna R., Sartini D., Emanuelli M., Mateuszuk L., Kij A., Chlopicki S., Escudé Martinez de Castilla P. (2021). Esterase-Sensitive Prodrugs of a Potent Bisubstrate Inhibitor of Nicotinamide N-Methyltransferase (NNMT) Display Cellular Activity. Biomolecules.

[99] van Haren M.J., Zhang Y., Thijssen V., Buijs N., Gao Y., Mateuszuk L., Fedak F.A., Kij A., Campagna R., Sartini D. (2021). Macrocyclic peptides as allosteric inhibitors of nicotinamide N-methyltransferase (NNMT). RSC Chem. Biol..

[100] Gao Y., van Haren M.J., Buijs N., Innocenti P., Zhang Y., Sartini D., Campagna R., Emanuelli M., Parsons R.B., Jespers W. (2021). Potent Inhibition of Nicotinamide N-Methyltransferase by Alkene-Linked Bisubstrate Mimics Bearing Electron Deficient Aromatics. J. Med. Chem..

[101] Bai J., Wang Z., Yang M., Xiang J., Liu Z. (2024). Disrupting CENP-N mediated SEPT9 methylation as a strategy to inhibit aerobic glycolysis and liver metastasis in colorectal cancer. Clin. Exp. Metastasis.

[102] Yang C., Wu J., He H., Liu H. (2020). Small molecule NSC1892 targets the CUL4A/4B-DDB1 interactions and causes impairment of CRL4(DCAF4) E3 ligases to inhibit colorectal cancer cell growth. Int. J. Biol. Sci..

[103] Li W., Zhou C., Yu L., Hou Z., Liu H., Kong L., Xu Y., He J., Lan J., Ou Q. (2024). Tumor-derived lactate promotes resistance to bevacizumab treatment by facilitating autophagy enhancer protein RUBCNL expression through histone H3 lysine 18 lactylation (H3K18la) in colorectal cancer. Autophagy.

[104] Peng W., Zeng Z. (2025). Epigenetic Activation of PTCD3 Promotes CRC Glutamine Metabolism and Metastasis via IGF2BP2-Mediated SLC38A2 m6A Modification. FASEB J. Off. Publ. Fed. Am. Soc. Exp. Biol..

[105] Liu J., Wang T., Zhang W., Huang Y., Wang X., Li Q. (2024). Association between Metabolic Reprogramming and Immune Regulation in Digestive Tract Tumors. Oncol. Res. Treat..

[106] Wang Y., Xu C., Yang X., Liu X., Guo Z., Lin X., Li L., Huang Z. (2024). Glycerol-3-phosphate acyltransferase 3-mediated lipid droplets accumulation confers chemoresistance of colorectal cancer. MedComm.

[107] Zi R., Zhao X., Liu L., Wang Y., Zhang R., Bian Z., Jiang H., Liu T., Sun Y., Peng H. (2025). Metabolic-Immune Suppression Mediated by the SIRT1-CX3CL1 Axis Induces Functional Enhancement of Regulatory T Cells in Colorectal Carcinoma. Adv. Sci..

[108] Du Z., Su J., Lin S., Chen T., Gao W., Wang M., Li Y., Wei D., Hu Z., Gao C. (2023). Hydroxyphenylpyruvate Dioxygenase Is a Metabolic Immune Checkpoint for UTX-deficient Colorectal Cancer. Gastroenterology.

[109] Zhang Z., Zheng Y., Chen Y., Yin Y., Chen Y., Chen Q., Hou Y., Shen S., Lv M., Wang T. (2022). Gut fungi enhances immunosuppressive function of myeloid-derived suppressor cells by activating PKM2-dependent glycolysis to promote colorectal tumorigenesis. Exp. Hematol. Oncol..

[110] Liu J., Zhang W., Chen L., Wang X., Mao X., Wu Z., Shi H., Qi H., Chen L., Huang Y. (2025). VSIG4 Promotes Tumour-Associated Macrophage M2 Polarization and Immune Escape in Colorectal Cancer via Fatty Acid Oxidation Pathway. Clin. Transl. Med..

[111] Liu S., Gong H., Li P., Hu J., Li Y., Xu R., Cai J., Wang S., Cai J., Ma H. (2025). Chemotherapy-induced macrophage CXCL7 expression drives tumor chemoresistance via the STAT1/PHGDH-serine metabolism axis and SAM paracrine feedback to M2 polarization. Cell Death Dis..

[112] Miao H., Ou J., Peng Y., Zhang X., Chen Y., Hao L., Xie G., Wang Z., Pang X., Ruan Z. (2016). Macrophage ABHD5 promotes colorectal cancer growth by suppressing spermidine production by SRM. Nat. Commun..

[113] Zhang D., Shi R., Xiang W., Kang X., Tang B., Li C., Gao L., Zhang X., Zhang L., Dai R. (2020). The Agpat4/LPA axis in colorectal cancer cells regulates antitumor responses via p38/p65 signaling in macrophages. Signal Transduct. Target. Ther..

[114] Meng X., Na R., Peng X., Li H., Ouyang W., Zhou W., You X., Li Y., Pu X., Zhang K. (2024). Musashi-2 potentiates colorectal cancer immune infiltration by regulating the post-translational modifications of HMGB1 to promote DCs maturation and migration. Cell Commun. Signal..

[115] Han B., He J., Chen Q., Yuan M., Zeng X., Li Y., Zeng Y., He M., Zhou Q., Feng D. (2023). ELFN1-AS1 promotes GDF15-mediated immune escape of colorectal cancer from NK cells by facilitating GCN5 and SND1 association. Discov. Oncol..

[116] Deng J., Li Y., Yin L., Liu S., Li Y., Liao W., Mu L., Luo X., Qin J. (2025). Histone lactylation enhances GCLC expression and thus promotes chemoresistance of colorectal cancer stem cells through inhibiting ferroptosis. Cell Death Dis..

[117] Zhu Y., Huang S., Chen S., Chen J., Wang Z., Wang Y., Zheng H. (2021). SOX2 promotes chemoresistance, cancer stem cells properties, and epithelial-mesenchymal transition by β-catenin and Beclin1/autophagy signaling in colorectal cancer. Cell Death Dis..

[118] Nishimura T., Nakata A., Chen X., Nishi K., Meguro-Horike M., Sasaki S., Kita K., Horike S.I., Saitoh K., Kato K. (2019). Cancer stem-like properties and gefitinib resistance are dependent on purine synthetic metabolism mediated by the mitochondrial enzyme MTHFD2. Oncogene.

[119] Ji Y., Yang C., Tang Z., Yang Y., Tian Y., Yao H., Zhu X., Zhang Z., Ji J., Zheng X. (2017). Adenylate kinase hCINAP determines self-renewal of colorectal cancer stem cells by facilitating LDHA phosphorylation. Nat. Commun..

[120] Liu S., Sun Y., Hou Y., Yang L., Wan X., Qin Y., Liu Y., Wang R., Zhu P., Teng Y. (2021). A novel lncRNA ROPM-mediated lipid metabolism governs breast cancer stem cell properties. J. Hematol. Oncol..

[121] Liu C., Wang J.L., Wu D.Z., Yuan Y.W., Xin L. (2022). Methionine restriction enhances the chemotherapeutic sensitivity of colorectal cancer stem cells by miR-320d/c-Myc axis. Mol. Cell. Biochem..

[122] Cui F., Chen Y., Wu X., Zhao W. (2024). Mesenchymal stem cell-derived exosomes carrying miR-486-5p inhibit glycolysis and cell stemness in colorectal cancer by targeting NEK2. BMC Cancer.

[123] Stintzing S., Wirapati P., Lenz H.J., Neureiter D., Fischer von Weikersthal L., Decker T., Kiani A., Kaiser F., Al-Batran S., Heintges T. (2019). Consensus molecular subgroups (CMS) of colorectal cancer (CRC) and first-line efficacy of FOLFIRI plus cetuximab or bevacizumab in the FIRE3 (AIO KRK-0306) trial. Ann. Oncol. Off. J. Eur. Soc. Med. Oncol..

[124] Mo S., Ye L., Wang D., Han L., Zhou S., Wang H., Dai W., Wang Y., Luo W., Wang R. (2023). Early Detection of Molecular Residual Disease and Risk Stratification for Stage I to III Colorectal Cancer via Circulating Tumor DNA Methylation. JAMA Oncol..

[125] Xie Z., Niu L., Zheng G., Du K., Dai S., Li R., Dan H., Duan L., Wu H., Ren G. (2023). Single-cell analysis unveils activation of mast cells in colorectal cancer microenvironment. Cell Biosci..

[126] Wang Y., Qiu X., Li Q., Qin J., Ye L., Zhang X., Huang X., Wen X., Wang Z., He W. (2025). Single-cell and spatial-resolved profiling reveals cancer-associated fibroblast heterogeneity in colorectal cancer metabolic subtypes. J. Transl. Med..

[127] Tao X.Y., Li Q.Q., Zeng Y. (2024). Clinical application of liquid biopsy in colorectal cancer: Detection, prediction, and treatment monitoring. Mol. Cancer.

[128] Ladabaum U., Mannalithara A., Weng Y., Schoen R.E., Dominitz J.A., Desai M., Lieberman D. (2024). Comparative Effectiveness and Cost-Effectiveness of Colorectal Cancer Screening with Blood-Based Biomarkers (Liquid Biopsy) vs Fecal Tests or Colonoscopy. Gastroenterology.

[129] Liang G., Zhu Y., Ali D.J., Tian T., Xu H., Si K., Sun B., Chen B., Xiao Z. (2020). Engineered exosomes for targeted co-delivery of miR-21 inhibitor and chemotherapeutics to reverse drug resistance in colon cancer. J. Nanobiotechnol..

[130] Zhou H., Zhu L., Song J., Wang G., Li P., Li W., Luo P., Sun X., Wu J., Liu Y. (2022). Liquid biopsy at the frontier of detection, prognosis and progression monitoring in colorectal cancer. Mol. Cancer.

[131] Du M., Gu D., Xin J., Peters U., Song M., Cai G., Li S., Ben S., Meng Y., Chu H. (2023). Integrated multi-omics approach to distinct molecular characterization and classification of early-onset colorectal cancer. Cell Rep. Med..

[132] Kamel F., Eltarhoni K., Nisar P., Soloviev M. (2022). Colorectal Cancer Diagnosis: The Obstacles We Face in Determining a Non-Invasive Test and Current Advances in Biomarker Detection. Cancers.

[133] Laussel C., Léon S. (2020). Cellular toxicity of the metabolic inhibitor 2-deoxyglucose and associated resistance mechanisms. Biochem. Pharmacol..

[134] Mu M., Zhang Q., Zhao C., Li X., Chen Z., Sun X., Yu J. (2023). 3-Bromopyruvate overcomes cetuximab resistance in human colorectal cancer cells by inducing autophagy-dependent ferroptosis. Cancer Gene Ther..

[135] Miyamoto R., Takigawa H., Yuge R., Shimizu D., Ariyoshi M., Otani R., Tsuboi A., Tanaka H., Yamashita K., Hiyama Y. (2024). Analysis of anti-tumor effect and mechanism of GLS1 inhibitor CB-839 in colorectal cancer using a stroma-abundant tumor model. Exp. Mol. Pathol..

[136] Fries B.D., Hummon A.B. (2024). FAS Inhibited Proteomics and Phosphoproteomics Profiling of Colorectal Cancer Spheroids Shows Activation of Ferroptotic Death Mechanism. J. Proteome Res..

[137] Chen L.Y., Wu D.S., Shen Y.A. (2024). Fatty acid synthase inhibitor cerulenin hinders liver cancer stem cell properties through FASN/APP axis as novel therapeutic strategies. J. Lipid Res..

[138] Tanaka K., Chamoto K., Saeki S., Hatae R., Ikematsu Y., Sakai K., Ando N., Sonomura K., Kojima S., Taketsuna M. (2022). Combination bezafibrate and nivolumab treatment of patients with advanced non-small cell lung cancer. Sci. Transl. Med..

[139] Laranjeira A.B.A., Nguyen D., Pelosof L.C., Doroshow J.H., Yang S.X. (2024). Upregulation of TET2 and Resistance to DNA Methyltransferase (DNMT) Inhibitors in DNMT1-Deleted Cancer Cells. Diseases.

[140] Huang K.C., Ke T.W., Lai C.Y., Hong W.Z., Chang H.Y., Lee C.Y., Wu C.H., Chiang S.F., Liang J.A., Chen J.Y. (2024). Inhibition of DNMTs increases neoantigen-reactive T-cell toxicity against microsatellite-stable colorectal cancer in combination with radiotherapy. Biomed. Pharmacother..

[141] Wei T.T., Lin Y.T., Tang S.P., Luo C.K., Tsai C.T., Shun C.T., Chen C.C. (2020). Metabolic targeting of HIF-1α potentiates the therapeutic efficacy of oxaliplatin in colorectal cancer. Oncogene.

[142] Humphreys K.J., Cobiac L., Le Leu R.K., Van der Hoek M.B., Michael M.Z. (2013). Histone deacetylase inhibition in colorectal cancer cells reveals competing roles for members of the oncogenic miR-17-92 cluster. Mol. Carcinog..

[143] Song Y., Ren S., Wu S., Liu W., Hu C., Feng S., Chen X., Tu R., Gao F. (2025). Glucocorticoid promotes metastasis of colorectal cancer via co-regulation of glucocorticoid receptor and TET2. Int. J. Cancer.

[144] Baretti M., Murphy A.G., Zahurak M., Gianino N., Parkinson R., Walker R., Lopez-Vidal T.Y., Zheng L., Rosner G., Ahuja N. (2023). A study of using epigenetic modulators to enhance response to pembrolizumab (MK-3475) in microsatellite stable advanced colorectal cancer. Clin. Epigenetics.

[145] Li C., Song J., Guo Z., Gong Y., Zhang T., Huang J., Cheng R., Yu X., Li Y., Chen L. (2022). EZH2 Inhibitors Suppress Colorectal Cancer by Regulating Macrophage Polarization in the Tumor Microenvironment. Front. Immunol..

[146] Jin Y., Cao Q., Chen C., Du X., Jin B., Pan J. (2015). Tenovin-6-mediated inhibition of SIRT1/2 induces apoptosis in acute lymphoblastic leukemia (ALL) cells and eliminates ALL stem/progenitor cells. BMC Cancer.

[147] Omar A., Govan D., Penny C. (2023). Epigenetic regulation in colorectal cancer: The susceptibility of microRNAs 145, 143 and 133b to DNA demethylation and histone deacetylase inhibitors. PLoS ONE.

[148] Huang X., Xu X., Ke H., Pan X., Ai J., Xie R., Lan G., Hu Y., Wu Y. (2022). microRNA-16-5p suppresses cell proliferation and angiogenesis in colorectal cancer by negatively regulating forkhead box K1 to block the PI3K/Akt/mTOR pathway. Eur. J. Histochem..

[149] Aziret M., Güney Eskiler G., Çakar G., Özkan A.D., Ercan M., Bilir C., Polat E., Koçer H.B., Yıldırım E.K., Duman M. (2023). Effect of the MiR-99b and MiR-135b on peritoneal carcinomatosis and liver metastasis in colorectal cancer. Clinics.

[150] Philip P.A., Buyse M.E., Alistar A.T., Rocha Lima C.M., Luther S., Pardee T.S., Van Cutsem E. (2019). A Phase III open-label trial to evaluate efficacy and safety of CPI-613 plus modified FOLFIRINOX (mFFX) versus FOLFIRINOX (FFX) in patients with metastatic adenocarcinoma of the pancreas. Future Oncol..

[151] Pujalte-Martin M., Belaïd A., Bost S., Kahi M., Peraldi P., Rouleau M., Mazure N.M., Bost F. (2024). Targeting cancer and immune cell metabolism with the complex I inhibitors metformin and IACS-010759. Mol. Oncol..

[152] Kuboki Y., Koyama T., Matsubara N., Naito Y., Kondo S., Harano K., Yonemori K., Yoh K., Gu Y., Mita T. (2024). PD-1 inhibition with retifanlimab and/or arginase inhibition with INCB001158 in Japanese patients with solid tumors: A phase I study. Cancer Med..

[153] Paskeh M.D.A., Saebfar H., Mahabady M.K., Orouei S., Hushmandi K., Entezari M., Hashemi M., Aref A.R., Hamblin M.R., Ang H.L. (2022). Overcoming doxorubicin resistance in cancer: siRNA-loaded nanoarchitectures for cancer gene therapy. Life Sci..

[154] Kang H., Ga Y.J., Kim S.H., Cho Y.H., Kim J.W., Kim C., Yeh J.Y. (2023). Small interfering RNA (siRNA)-based therapeutic applications against viruses: Principles, potential, and challenges. J. Biomed. Sci..

[155] Sendi H., Yazdimamaghani M., Hu M., Sultanpuram N., Wang J., Moody A.S., McCabe E., Zhang J., Graboski A., Li L. (2022). Nanoparticle Delivery of miR-122 Inhibits Colorectal Cancer Liver Metastasis. Cancer Res..

[156] Jung J.W., Kim J.E., Kim E., Lee H., Lee H., Shin E.A., Lee J.W. (2022). Liver-originated small extracellular vesicles with TM4SF5 target brown adipose tissue for homeostatic glucose clearance. J. Extracell. Vesicles.

[157] Danaei M., Dehghankhold M., Ataei S., Hasanzadeh Davarani F., Javanmard R., Dokhani A., Khorasani S., Mozafari M.R. (2018). Impact of Particle Size and Polydispersity Index on the Clinical Applications of Lipidic Nanocarrier Systems. Pharmaceutics.

[158] Schlegel M.K., Matsuda S., Brown C.R., Harp J.M., Barry J.D., Berman D., Castoreno A., Schofield S., Szeto J., Manoharan M. (2021). Overcoming GNA/RNA base-pairing limitations using isonucleotides improves the pharmacodynamic activity of ESC+ GalNAc-siRNAs. Nucleic Acids Res..

